# Multicolor Phenylenediamine Carbon Dots for Metal-Ion Detection with Picomolar Sensitivity

**DOI:** 10.1021/acsanm.1c02496

**Published:** 2021-09-08

**Authors:** Hani Barhum, Tmiron Alon, Mohammed Attrash, Andrey Machnev, Ivan Shishkin, Pavel Ginzburg

**Affiliations:** † Department of Physical Electronics, Electrical Engineering, Ramat Aviv, Tel Aviv 69978, Israel; ‡ Light-Matter Interaction Centre, Tel Aviv University, Tel Aviv 69978, Israel; § Schulich Faculty of Chemistry, Technion—Israel Institute of Technology, Haifa 32000, Israel; ∥ Center for Photonics and 2D Materials, Moscow Institute of Physics and Technology, Dolgoprudny 141700, Russia

**Keywords:** carbonization, fluorescence, quantum yield (QY), sensing, molecular dynamics (MD)

## Abstract

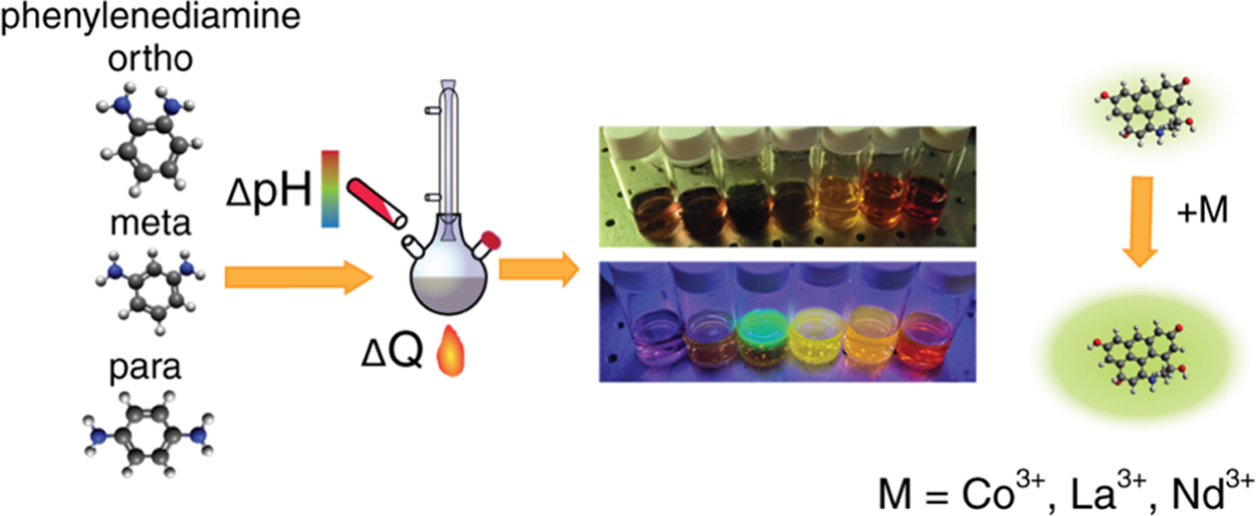

Carbon dots keep attracting attention in multidisciplinary fields, motivating the development of new compounds. Phenylenediamine C_6_H_4_(NH_2_)_2_ dots are known to exhibit colorful emission, which depends on size, composition, and the functional surface groups, forming those structures. While quite a few fabrication protocols have been developed, the quantum yield of phenylenediamine dots still does not exceed 50% owing to undesired fragment formation during carbonization. Here, we demonstrate that an ethylene glycol-based environment allows obtaining multicolor high-quantum-yield phenylenediamine carbon dots. In particular, a kinetic realization of solvothermal synthesis in acidic environments enhances carbonization reaction yield for meta phenylenediamine compounds and leads to quantum yields, exciting 60%. Reaction yield after the product’s purification approaches 90%. Furthermore, proximity of metal ions (Nd^3+^, Co^3+^, La^3+^) can either enhance or quench the emission, depending on the concentration. Optical monitoring of the solution allows performing an accurate detection of ions at picomolar concentrations. An atomistic model of carbon dots was developed to confirm that the functional surface group positioning within the molecular structure has a major impact on dots’ physicochemical properties. The high performance of new carbon dots paves the way toward their integration in numerous applications, including imaging, sensing, and therapeutics.

## 1 Introduction

Carbon dots (CDs), which emerged as side products in a single-walled carbon nanotube assembly,^1^ are an expanding field for investigation owing to their low-cost facile fabrication^2,3^ and quite a few remarkable properties.^4^ Phenylenediamine (PD) CDs already serve numerous practical applications, acting as sensing agents for metal ions,^5^ active materials for light-emitting diodes,^6^ cancer phototherapy agents,^7^ bioimagers,^8^ polarity, and pH indicators.^9^ CDs have uniform size distributions with means below 10 nm and demonstrate high aqueous solubility, high sensitivity to the chemical environment, low toxicity, good biocompatibility, and tunable photoluminescence (PL) along with chemical stability and photostability to name just a few.^2^ As a result, new CD fabrication techniques, aiming to achieve improved properties, keep attracting attention.

Synthesis techniques at a high level can be separated into top-down and bottom-up approaches. The bottom-up methods have several advantages, including lower cost, scaling-up capabilities, improved size uniformity, and higher quantum yield of the product, according to recent reports.^10^ Solvothermal synthesis, being applied at relatively mild conditions, is one of the simplest facile approaches, allowing one to obtain different fluorescent properties.^11^ The majority of CDs emit light in the blue spectral range, and a few CDs were found to emit red light. Nitrogen (N), phosphorus (P), and oxygen (O) dopants cause a spectral shift, pushing emission lines to green and yellow.^7,12^ Furthermore, the fluorescence properties of the reaction products can be controlled by changing reaction conditions in bottom-up approaches. The doping is achieved with an organic precursor, by changing reaction conditions, and by adding another rich source of dopant atoms.^13^ Thus, the CD emission spectrum can be manipulated with a combination of different factors, including carbon core size, core atomic composition, molecular ring types, and side groups functionalizing the CD structure.^14^

Emission and absorption spectra are the main characteristics of fluorescent materials along with their quantum yields, which are always subject to maximization. Carbon-based phenylenediamine derivatives, i.e., “para,” “meta,” and “ortho,” being fabricated by solvothermal processes in ethanol, demonstrate superior light-emitting properties over the entire visible spectrum.^8^ Fluorescence in PD CD materials is dominated by N–O doping. Traditionally, a strong acid hydrothermal treatment is employed for obtaining doped CDs.^13,15^ Previously, PD CDs were demonstrated to provide yellow emission around 550 nm, shifting to 600 nm red emission owing to P doping within the phosphoric acid environment.^7^ The chemical properties of doped CDs strongly depend on side and core groups, positioned on the molecules. The diversified functionalities originate from the spectacular chemical properties, provided by dopant atoms.^14^

Despite numerous applications of PD CDs, their synthetic methods can still be significantly improved.^11^ Moreover, traditional fabrication methods using Teflon-lined autoclave lead to a rather limited fluorescence quantum yield. In most of the reports, the values approach 10%, while more sophisticated protocols succeeded in reaching 20–30% quantum yield, while only a few recent demonstrations have crossed 50%.^14^ Finding new pathways to increase quantum yields of PD CDs can further extend ranges of their practical applicability and integrate these materials in a widely used technology. Furthermore, reaction yield (RY) is an additional crucial parameter. In most of the common synthesis methods, hydrothermal and solvothermal RYs are significantly lower than 2%.^11^

**Figure 1:**
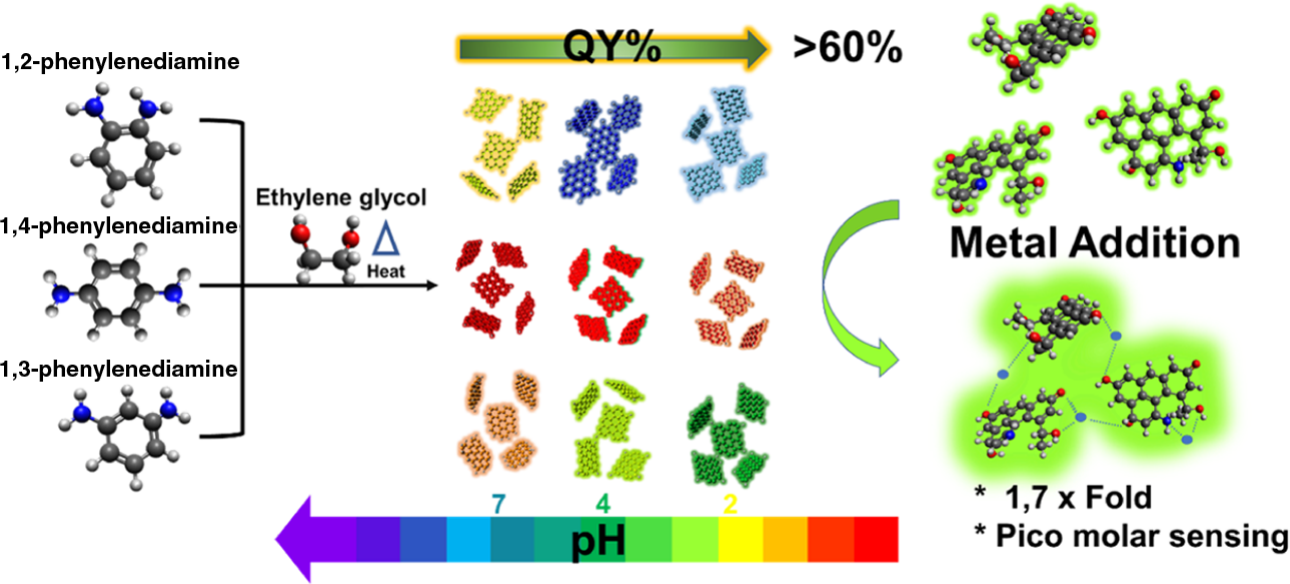
Graphical abstract. (Left) Molecular representation of the phenylenediamine isomers with ethylene glycol. (Middle) Colorful products, obtained at different pH levels for each isomer. pH, indicated with a colored arrow at the bottom, is the main kinetic parameter for the reaction control. The top arrow represents the QY increase, correlated with the pH drop. (Right) Schematic of the metal interaction with *m*CDs, leading to the photoluminescence increase or quenching, depending on the ionic concentration.

To optimize RYs, key parameters governing CD formation dynamics should be investigated. Kinetic studies of carbonization are still overlooked, except for a few basic studies.^16^ In this work, a systematic investigation of the carbonization reaction parameters of PD in ethylene glycol (EG) via a solvothermal method is made. New insights into optical tunability of emission and absorbance of phenylenediamine isomers with an emphasis on meta isomer carbon dots (*m*CDs) are developed. The parameters of reaction time, acidity, PD initial concentration, temperature, and viscosity are investigated on pathways to explore colorful products with an emphasis on optimizing reaction conditions for the highest RY and QY of the product. Furthermore, the time-monitored dynamics of carbonization is comprehensively studied and fitted with a basic consecutive first-order model. The immediate outcome is the acid role in the process and its direct impact on QY and RY. The analysis of acidity of the reaction bath revealed hidden properties, related to the dynamical control of carbonization. Analytic and qualitative techniques, including high-performance liquid chromatography (HPLC), X-ray photoelectron spectroscopy (XPS), and liquid chromatography-mass spectrometry (LCMS), provide new insights into the carbonization process, allowing one to analyze RY and supply further information on the chemical properties. The molecular structure of reaction products is then confirmed with CP2K software simulations. The modeling provides an estimated molecular configuration, including electronic structure, linked to the observed experimental absorption and fluorescence spectra. Metal sensing in biological conditions is demonstrated in a phosphate-buffered saline (PBS) solution. Metal-enhancement fluorescence was observed at subnanomolar ion concentrations, paving the way to sensing applications (Figure 1).

## 2 Experimental Section

### 2.1 Phenylenediamine CD Synthesis

All chemicals were purchased from Merck Ltd. Synthesis of CDs was performed in a refluxed solvothermal reaction. Briefly, 10 mg/mL PD isomer reacted in EG. To study the formation kinetics, the different parameters were tuned, including environmental temperature, viscosity, and acidity. Reaction times and additive compositions were varied. The growth protocol was implemented in a reflux apparatus and on a compatible heating plate in a scintillation vial of 20 mL. Table S1 summarizes reactions, which were investigated. The plate temperature was set to 453 K, unless otherwise noted.

### 2.2 Optical Characterization

Photoluminescence excitation spectroscopy (PLE) was performed with a plate reader SynergyH1. Absorbance spectra were obtained with a Macys1100 spectrophotometer with a tungsten lamp source and a silicon photodiode detector. The spectral band was set to 2 nm with an accuracy of 1 nm. Samples were diluted with deionized water before tests. Fluorescent lifetime measurements were done with a PicoQuant system, which uses the Taiko picosecond diode as a 375 nm excitation. The detailed methodology is presented in the Supporting Information.

### 2.3 Structural Characterization of Particles

The CD particles were purified and then analyzed with XPS. Size distributions were determined with transmission electron microscopy (TEM) at 200 kV acceleration voltage (a small fraction was imaged) and confocal dynamic light scattering (DLS). ζ potential was measured with the Zetasizer Nano ZS. HPLC was used to determine RY, and LCMS was used to follow reaction fragment dynamics. All of the methodologies are elaborated in the Supporting Information.

### 2.4 Quantum Yield and Reaction Yield

Quantum yield measurements were performed in a Horiba Jobin Yvon FL3-11 spectrofluorometer and a SynergyH1. QY was assessed by comparing to a known standard using a slope method for statistical results as follows: , where the slope refers to the absorbance vs fluorescence curve, ⌀_*x*_ is the QY under study, ⌀_*r*_ is a reference QY, and  is the ratio of refractive indices of the solvents. Fluorescein in 0.1 M NaOH in DIW was used as a reference and further compared versus Rhodamine 6G to double-check the accuracy. RY was determined by the product weight after purification and verified with HPLC. The reaction extract at each point was weighted, and the reaction volume was monitored. The changes in volume and extracted molecules were used to measure the RY. More details can be found in the Supporting Information.

### 2.5 Theoretical Section

The calculations in this study were performed by the CP2K code,^17^ which uses a Gaussian basis set complemented by an auxiliary plane-wave basis. Structures were drawn by the VESTA code.^18^ We used a triple-ζ polarization quality Gaussian basis set (TZVP-MOLOPT-GTH)^19^ and a 300 Ry plane-wave cutoff. Geometry optimization of the samples was performed by the Broyden–Fletcher–Goldfarb–Shanno (BFGS) routine using the Gaussian plane-wave (GPW) method with the combined density functional of B88 (exchange functional) and LYP^20,21^ (correlation functional), where all atoms were relaxed until the residual force was smaller than 0.05 eV/Å. We applied a structural minimization algorithm using a periodic model in the Γ-point approximation. The electronic structure was investigated using the hybrid functional B3LYP.^22^

## 3 Results and Discussion

It is remarkable but not surprising that the different derivatives of the same material result in dissimilar CD properties. Since quite a few parameters might affect RY and final products’ optical properties (such as QY), the key contributors to multicolor emission, tailored by the EG solvothermal environment, have to be identified first. To retrieve those, each reaction product (at least the main ones) has to be chemically identified and quantified. In our case, it was done using optical characterization techniques. In particular, the amine group’s relative positions on the benzene ring were found to play a key role in PL of yielded CD. As a result, relevant CD concentrations can be straightforwardly quantified with the aid of conventional spectroscopic tools, which are elaborated in detail in Section 3.5 and in the Supporting information. Moreover, the emission intensity dynamics points to changes occurring in the reactor. Therefore, there is a continuous transformation between photoactive and inactive CDs and other chemical species in a thermal process. The collision between the chemical species in the reactor results in a different product that can be controlled kinetically and thermodynamically, as will be elaborated in Sections 3.1–3.3.

### 3.1 Solvothermal Synthesis and Optical Characterization

**Figure 2:**
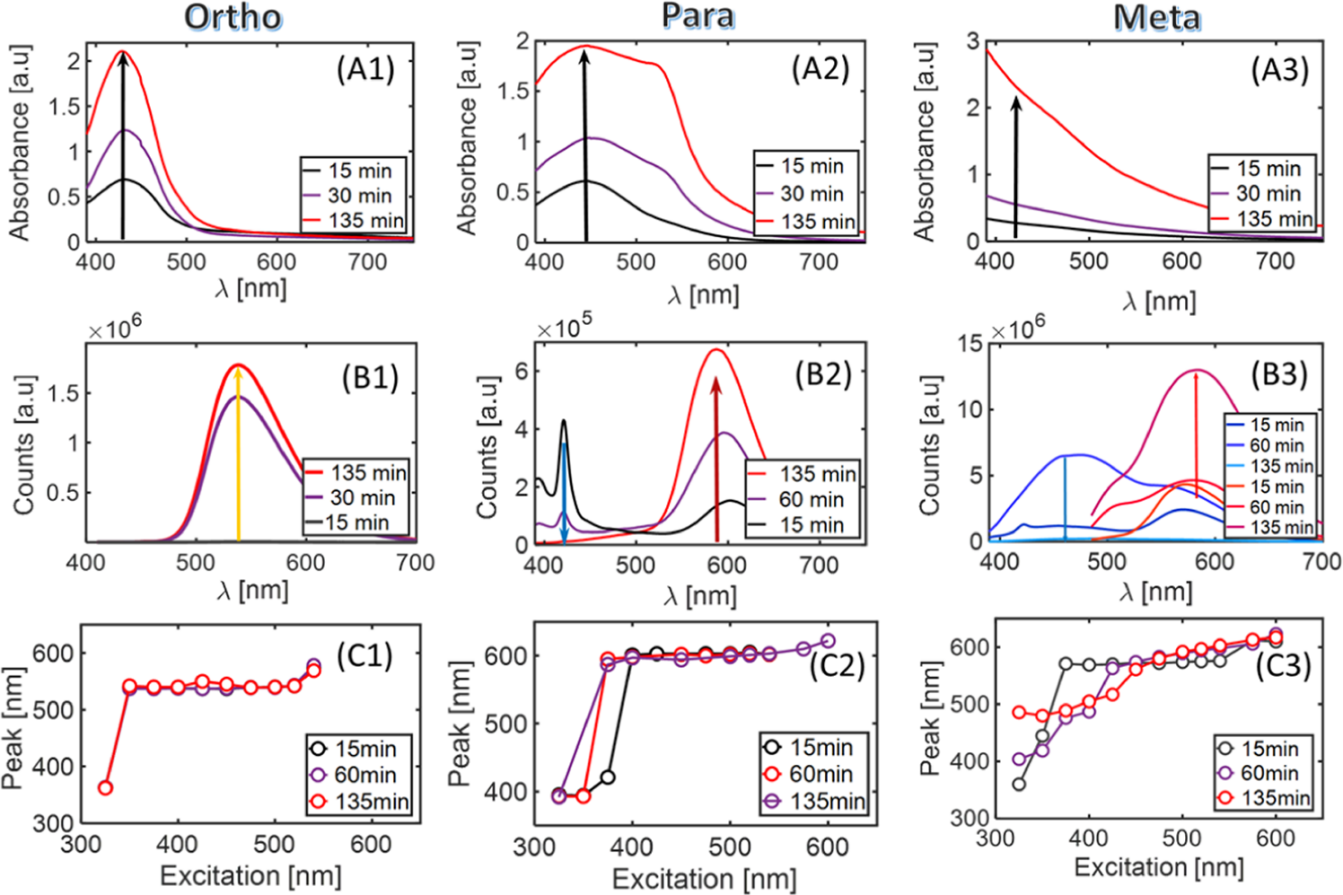
(A1–A3) UV–vis absorbance spectra of ortho, para, and meta PD CDs of αCDs synthesized in a solvothermal process in pure EG. Black, purple, and red lines correspond to 15 min, 30 min, and 135 min of reaction, respectively. (1) *o*-αCDs. (2) *p*-αCDs. (3) *m*-αCDs. (B1–B3) Emission spectra of carbonized products, obtained in pure EG. *o*-αCDs excited at 375 nm, *p*-αCDs excited at 375 nm, and *m*-αCDs excited at 475 nm (red) and 375 nm (blue). (C1–C3) Emission peak location as a function of the excitation wavelength.

The first reaction set was performed in a pure EG solvent (Supporting Information, Table S1, first row). In particular, PD isomers with a concentration of ∼10 mg/mL in EG were heated to 453 K with no additional chemicals in a refluxed system. Reaction products are monitored at different time slots and linked to optical properties and RY. The collected samples from the reaction are cooled to room temperature and stored in a dark container. The measurements on the samples are taken after 24 h. No significant changes were recorded in the optical properties of the collected samples within up to six months after collection. Figure 2 summarizes the detailed analysis of absorbance and excitation-dependent emission dynamics evolution of the products over the reaction time.

The absorbance spectra for all three isomers are shown in Figure 2A1–A3. Reaction *o*-α shows a gradual increase in absorption over time. The graph has a single peak around 440 nm (Figure 2A1). *p*-αCDs demonstrate one absorption peak around 450 nm at the beginning of the reaction (∼15 min), while another overlapping peak at 550 nm appears and leads to an absorbance band in the range of 400–600 nm (Figure 2A2). *m*-α CD absorbance has no local maxima in the optical range. The absorbance increases at shorter wavelengths over the reaction time (Figure 2A3).

The evolution of fluorescence over time was assessed for all isomer products (Figure 2B1–B3). Excitation of 375 nm was used. An increase in peak intensity at 550 nm is observed over the reaction time for *o*-αCDs, which is consistent with the increase in absorbance (Figure 2B1). In cases of *p*-α**-** and *m*-α**-**CDs, two peaks appear in emission spectra—one band emerges around 420–450 nm (“blue”), while another is located around 600 nm (“red”). Red-shift effects are attributed to the formation of bigger and oxygen-/nitrogen-doped carbon dots that attain red emission. Fluorescence intensity of the “blue” band decreases over the reaction time for both *p*-α**-** and *m*-α**-**CDs, while the intensity of the “red” band increases (Figure 2B2,B3). In the case of *m*-α**-**CDs, detecting fluorescence under 375 nm excitation becomes challenging owing to the strong reabsorption of light in the sample; thus, an additional set of spectra under 475 nm excitation is shown in panel B3.

At the next stage, each product was pumped with a tunable laser, scanning the excitation wavelength between 320 and 600 nm. The results are presented in panels C1–C3, whereas the full data set of spectra appears in the Supporting Information (Figure S1). In the case of *o*-α CDs, no peak position shift was observed for wavelengths from 350 to 540 nm for all samples collected at 15, 60, and 135 min. Thus, optical properties of *o*-αCDs are identical at different reaction times (Figure 2C1). The *p*-α CD excitation PLE scans show emission spectra that contain less blue fraction at 325–375 nm concerning reaction progress, leaving one main emission peak at 600 nm (Figure 2C2). The spectral shift of the emission band depends on the excitation wavelength, and this behavior is more pronounced in *m*-α CDs compared to the other two samples. At the beginning of the reaction, the position of maximum emission intensity shifts from UV up to 580 nm. The slope of the peak position vs excitation curve decreases with time and shows quite a broad range of tunabilities. This shift correlates with the evolution in the absorbance spectra (Figure 2C3). For the *p*-α CDs, an increase in intensity of the emission band around 600 nm was observed over the increase in the reaction time. Detailed photoluminescence spectra under different excitation wavelengths (325–625 nm) at 25 nm steps for the whole set of (*p*-*o*-*m*)-α CDs are presented in Figure S1.

### 3.2 Initial Acidity Effect

Endeavors to control emission properties drew our attention to a set of parameters involved in the reaction, namely, solvent type, acidity, viscosity, and concentration of PD. The parameterization will be introduced into a global kinetic (RY-QY) model and then subsequently optimized in terms of kinetic constants.

In the PD case, side group position plays a crucial role in the reaction evolution that ends up with a specific product. Protonated/deprotonated side groups, in our case amines, are altered through the acidity of the medium at the beginning of the reaction. The presence of an extra proton on PD side groups prevents the oxidation of the positively charged amine group by shielding the unpaired electrons of the nitrogen atom. Otherwise, fast oxidation from amine to nitro group results in a quick degradation (relative to formation rate) of the active CDs, along with the reduction of the fluorescent product RY. Acidity is a known key parameter to control the reaction output and its stability over time. The shielded amine group reacts with another PD ring to create CDs. Using EG as a reaction host medium (e.g., increase in viscosity and suppression of diffusion speed) and variation in acidity, it was possible to control reaction rates and reduce the product oxidation speed, thus allowing controllable product formation. Overoxidation leads to a loss of fluorescence efficiency owing to the formation of a larger fraction of inactive molecular species. Virtually, the initially transparent solution of PD turns into a yellow-colored suspension due to oxidation of the amine into nitro groups (Figure 5). Thus, the behavior of CD formation reactions should differ between seed PD isomers, which would result in discrepancies in absorption and emission properties of reaction products with changes in acidity. The latter parameter was controlled by introducing either HCl or KOH to the initial solution of PD precursors, as it is described in the experimental section.

**Figure 3:**
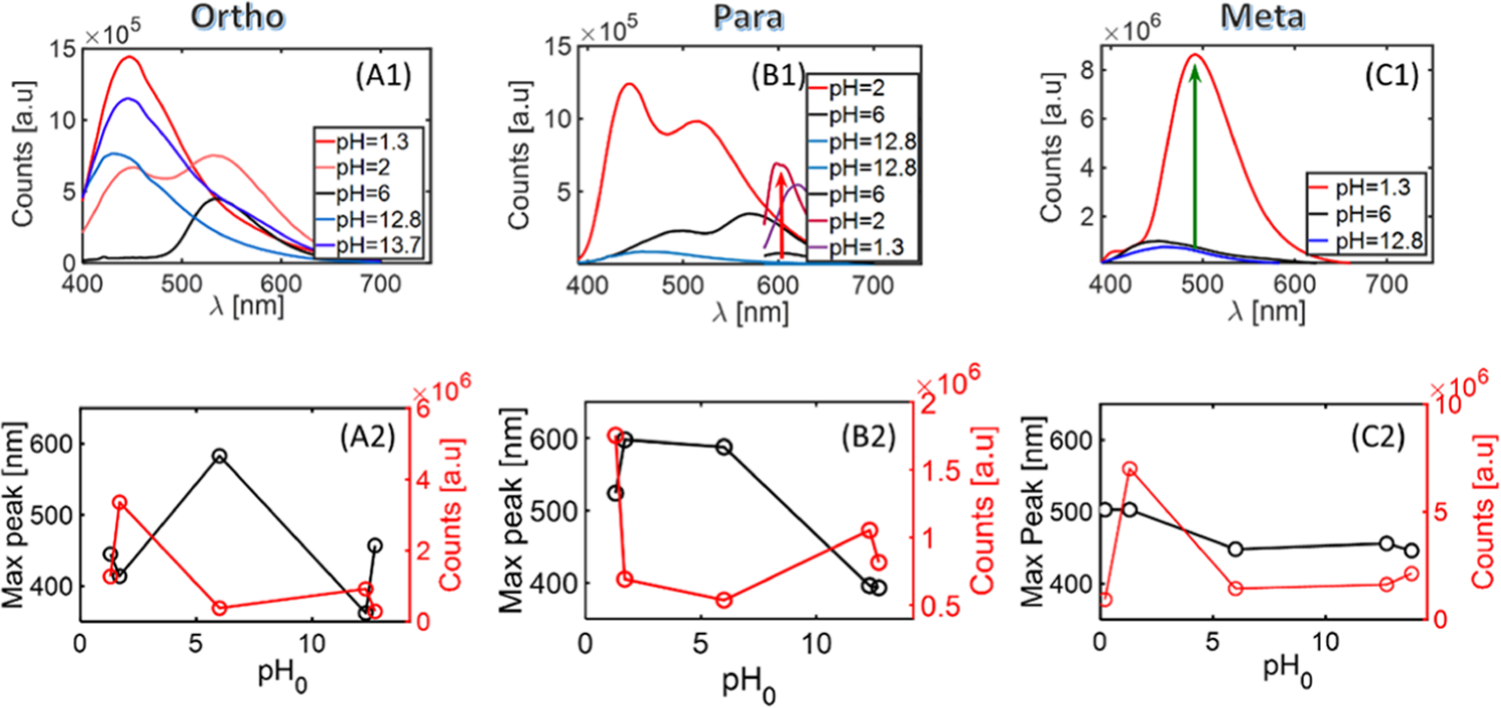
Emission spectra of PD CDs, prepared in different acidities. First row: red lines, acidic; black line, neutral; blue line, basic. Second row: black lines, peak position; red lines, peak intensity. (A1) *o*-βCDs excited at 375 nm. (B1) *p*-βCDs excited at 375 nm. The arrows mark the intensity gradient in the basic to acidic carbonization process. (C1) *m*-βCD emission in blue and red upon excitation of 375 and 575 nm, respectively. (A2), (B2), and (C2) correspond to *o*-βCDs, *p*-βCDs, and *m*-βCDs, respectively. Peak position (left *y*-axes), and intensity in arbitrary units (right *y*-axis) vs the initial pH.

The analysis of CD emission properties on reaction medium acidity is summarized in Figure 3. In the case of *ortho*-PD isomers (reactions *o*-β), the emission peak intensity increases at 450 nm, as was observed in both mild basic and acidic reactions. However, PL intensity drop was observed when the concentration of the acid/base was further increased. The emission peak position in neutral and mild acidic reactions appeared around 550–600 nm and then shifted to 450 nm (Figure 3A).

*p*-β1CDs showed more intense emission as a consequence of acidification. An increase in acidity results in a significant rise in RY and QY of reaction products. It should also be noted that the green emission band centered at 520 nm appears in acidic environments and becomes dominant in the emission spectra with an increase in acidity. We found that the reaction products have a predominantly red emission at 610 nm from *p*-βCDs with an optimal acidity of 0.013 M for para isomers. Further increase in acidity results in a green emission band around 520 nm becoming dominant at 0.066 M acid concentrations and higher, while blue emission dominates the basic region (Figure 3B).

The meta isomer (*m*-β CDs) CD synthesis resulted in green emission around 500 nm for the acidic reaction and blue (around 450 nm) for a basic reaction. The peak intensity increases with an increase in acidity of the environment (Figure 3C).

As expected, PD carbonization in the basic environment (β4-5 reaction set) resulted in the formation of oxidized species and CDs with blue emission around 450 nm for all isomers. The low values of RY < 10% and QY < 1% of reactions in the basic environment are consistent with rapid oxidation of amine groups and low efficiency of active CD formation. On the other hand, acidic reactions resulted in a higher efficiency (in terms of RY and QY) for all CDs. The main peak position shifted to 500 nm for all of the products formed in an acidic environment.

Temperature sweep investigation is then made for the reaction set β products, referred to in Table S1 as set β a-b-c with 453–513–573 K, respectively. The initial acidity impact on the final product properties is consistent through the set (Figures S2–S4), where the acidic environment boosts the RY/QY of *m*,*p*-βCDs with a shift in emission to 500–530 nm (Figure 3B2–C2). Hence, concentrated hydrochloric acid addition investigation is carried out in the following set to realize the impact on CDs. Acid molarity increases RY and QY; thus, we investigated more acidic reactions to find the optimal concentration.

In the γ reaction set, 12 M hydrochloric acid was introduced to the reaction bath to examine the limits of the acidity enhancement. Concentrated hydrochloric acid as a carbonization enhancer introduced into the EG medium at 10 v/v% resulted in brighter dots at 420–450 nm excitation (Figure S5A–C). Intensity over time shows the nonmonotonic behavior of the luminescent CD fraction. The fluorescent CD fraction (e.g., RY) reaches the peak value at a certain moment of time after the reaction was initiated, which could be explained by simultaneously occurring processes of CD generation and degradation. Different PD isomers reach maximal fluorescence intensity at different times: *p*-CDs at 3 h from the beginning of reaction, mCDs at 2 h, and *o*-CDs at 4 h. This behavior can be attributed to the thermal degradation of fluorescent CDs (Figure S7). Saturation in emission appears in *p*-γ CDs after 48 h, *o*-γ CDs after 10 h, and *m*-γ CDs after 3 h. Furthermore, PLE spectra revealed that *m*-γ CDs contain two distinct absorption bands, centered around 370 and 500 nm (Figure S6B), with similar emission intensities both contributing to the 530 nm emission band (Figure S5B). The *p*-γCD emission appears around 560 nm (Figure S5A), as expected from previous results. The red emission band intensity decreases relative to the emission at 560 nm. PLE spectra of *p*-γCDs show broadband absorption related to the emission at 560 nm (Figure S6C,D). *o*-γCDs drastically change their optical properties within the reaction time. The increase in fluorescence intensity is then followed by a rapid drop during reaction and accompanied by a shift of the emission peak to the blue region around 420–450 nm (Figure S5C). Our proposed hypothesis suggests that the creation of CD is initialized in a core fluorescent molecule. The latter is subjected to chemical changes that are a consequence of the molecular environment and reaction parameters. The driving energetic terms are the thermokinetics that favors certain molecular trajectories affected by main parameters, in particular carbon source, pH, real-time concentration, and temperature. The kinetic control will be elaborated in Section 3.5.

However, carbonization of both meta and para isomers has demonstrated a response to acidification in terms of emission intensity (Figure S8). To establish the correct choice of protonating acid, a set of carbonization reactions for the *m*PD isomer using different acids with identical concentrations was performed (reactions δ1−δ5). *m*CDs with the highest emission intensity around 500 nm were obtained using hydrochloric acid (δ1 reaction). Diprotic sulfuric acid with 2.4 M concentration in the case of 10% v/v was found to be the second-most efficient additive δ2 (Figure S8A). Lower emission intensities were observed for reactions δ3-5 compared to δ1,2. On the other hand, *m*-δ3CDs had a relatively high QY > 30%. The emission spectra were identical for all acids used (Figure S8A), resulting in the appearance of broad emission peaks around 510 nm. The QY value of the product δ1, assembled in hydrochloric acid, is the highest (45%) after 24 h. Therefore, we chose hydrochloric acid as the optimum for our systematic study.

### 3.3 Viscosity Effect

A solvent’s viscosity affects the reaction rates and can be controlled by replacing the EG fraction (90%) with viscous poly(ethylene glycol) (PEG). Furthermore, the media replacement maintains approximately the same chemical properties in terms of solvent reactivity or solvent–PD molecular interaction.

Higher solvent viscosity reduces the molecular mobility of reaction components; thus, the Arrhenius model of reaction kinetics suggests lower product conversion. The lowest viscosity of EG compared to PEG results in the highest carbonization reaction output for EG used as the reaction medium. The impact of viscosity on carbonization reaction is not underlined here and will be discussed qualitatively in the Supporting Information (Figure S9A–C). Yet, it is worth noting that the main differences were determined in the fluorescence intensity of the end product, which is directly linked to RY. Furthermore, the purification of ζCDs from PEG is easier and more efficient compared to EG due to the relatively higher diversity of chemical properties of active and inactive end products.

### 3.4 Fluorescent Lifetime

Decay dynamics of the products were investigated (Figure S11). The subsequent analysis shows the appearance of two characteristic lifetimes. Averaged lifetimes are less than 3 ns for *m*-ηCDs, 4 ns for *p*-ηCDs, and 7 ns for *o*-ηCDs, which shows the slowest decay (Figure S10d).

### 3.5 Molecular Properties

In this section, we reveal an approximated structure of our CDs through their chemical characteristics by performing employed electron-beam (TEM and XPS), chromatographic methods (LCMS), and IR spectroscopy.

#### 3.5.1 Liquid-Phase Chromatography Mass-Spectrometry

Liquid-phase chromatography mass-spectroscopy provides an insight into the molecular weight and size of the reaction products. The mass spectra of reaction solutions were taken after different reaction times to analyze products’ mass distributions. Additional tests were performed two months after synthesis and showed no measurable differences in product composition.

CDs mass spectra (MS) measurements from initially protonated PD precursors are consistent with fraction increase (mass and counts) and are shown in Figure S13. At the end of the reactions (Figure S13), unprotonated PD leftovers can be identified as a peak shift from 109 to 107 a.m.u, indicating that protons participate in the reaction. An overview of the LCMS spectra (Figure S13) reveals the presence of additional peaks in the mass spectrum, which can be attributed to the appearance of carbonization products. The mass distribution of the products shifts to heavier masses around 200 to 500 a.m.u. for the reactions for all isomers, which is consistent with the spectroscopic data. These mass values should correspond to average CD sizes of 3–4 nm.

#### 3.5.2 Transmission Electron Microscopy and Dynamic Light Scattering

**Figure 4:**
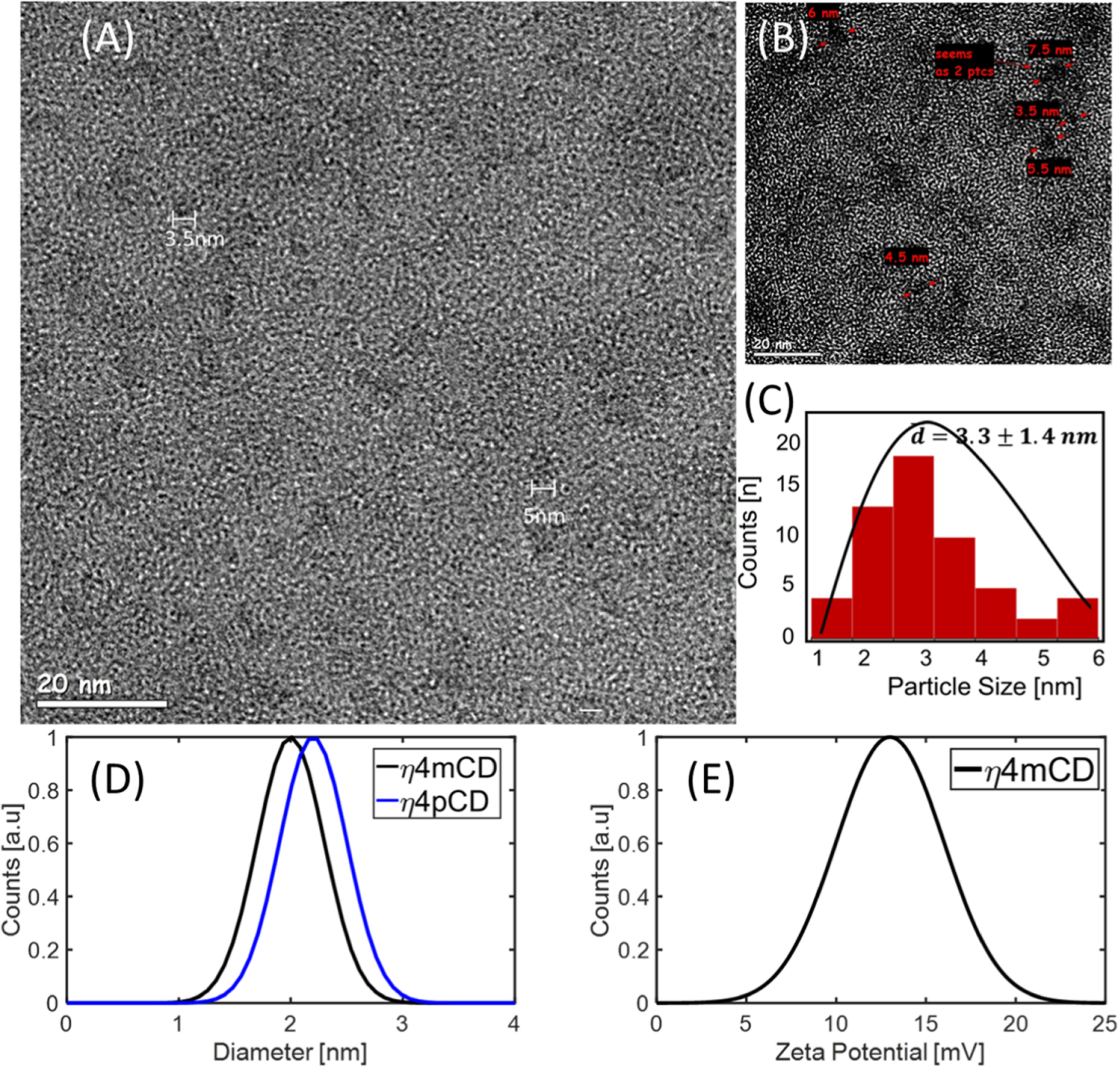
(A) HRTEM image of *m*CDs, dried over a carbon copper grid; scale bar is 20 nm. (B) Zoomed image—arrows pointing at the diameter of single dots. (C) Size distribution of particles in HRTEM. (D) DLS spectra of mη4-pη6CDs with a peak value around 2 nm. (E) ζ potential of *m*-η4CDs.

The qualitative data on the carbonization reaction products are further supported by DLS and TEM. A TEM image of the cluster of the CDs is shown in Figure 4A,B. Individual CDs can be seen with their average sizes of 2–5 nm (Figure 4C), which is consistent with LCMS. The CD size distribution was also analyzed using DLS, showing a peak around 2 nm for all CDs. The anisotropic geometry of the particles results in averaging over the angles of rotation for the molecules, and the value is expected to be smaller than the actual diameter. ζ potential is positive within a range of 15–25 mV (Figure 4D,E).

#### 3.5.3 X-ray Photoelectron Spectroscopy

XPS measurements were conducted to analyze the differences between the reaction products of different isomers. For this analysis, CDs were drop-casted and dried on a SiO_2_ substrate. The recorded spectra for all three isomers are presented in Figure S14. According to the analysis, the material is composed mainly of carbon atoms. The suggested empirical formula for *m*CDs that arises from the XPS is 1:4:20 N/O/C ratio, respectively. *m*CDs mainly consist of carbon, which is very logical in terms of the CD molecular skeletal composition, and less nitrogen/oxygen content. Furthermore, the CD oxygen content is concluded from both XPS data and FTIR. We hypothesize that oxygen doping is initialized with the exchange of amine side groups of PD and from the well-established method of ethylene glycol surface passivation.^23^ Thus, enhancement of the formation of carboxyl and carbonyl groups at the CD surface appeared by deconvoluting C 1s–O1 s peaks. The main atom attached to the forming molecules is oxygen and then nitrogen in lower amounts. XPS data also reveals that the main bonds formed in *m*CDs are carboxyl/carbonyl groups. As for N 1s peak deconvolution, it is mainly integrated in a pyridinic ring and then in a pyrrolic ring and integrated as graphitic nitrogen in *p*,*m*CDs^24,25^ (Figure S14C1,C2).

Here, we assume that carbonization source isomers are partially oxidized; thus, the resulting CDs would consist only of C, N, and O atoms. XPS elemental fragmentation analysis allowed us to establish component ratios by direct peak integration for each CD source isomer with *m*-γCDs having the highest carbon content around 78.7%. *o*-γCDs and *p*-γCDs contain similar quantities of carbon (around 75%), while oxygen found in *p*-γCDs is the highest (18.7% compared to 15.5–15.7% in cases of o- and mCDs).

Numerous studies suggested that the CD fluorescence red-shift originates from its degree of surface oxidation. It was shown^26^ that the CD emission red-shift correlates with the oxygen content on the CD surface with a higher degree of oxidation corresponding to larger quantities of surface traps.^14,27^ Trapping of excitons on oxygen defects results in longer radiative lifetimes as well as in red-shifted emission owing to lower energy gap values of defect states. Studies show that the linear relation between purified carbonization fragments with a red shift correlates linearly with the degree of oxidation.^27^ Thus, a higher degree of oxidation results in higher amounts of traps for excitons. On the other hand, band-gap analysis shows a strong link to oxygen content, in contrast to surface oxidation.^15,26,28^

Higher oxygen content in *p*-γCDs compared to the other two isomers supports the oxygen doping hypothesis of CDs, which is consistent with the emission spectra red shift.^7,24,29^ Nitrogen content is the lowest in *m*-γCDs, while in **o**,*p*-γCDs, it has similar nitrogen abundance (Table S4).

#### 3.5.4 Fourier Transform Infrared Spectroscopy

Fourier transform infrared (FTIR) spectroscopy allows characterizing chemical bonds in CDs. This method allows unrevealing the presence of actual functional chemical groups, which can correspond to fluorescence. FTIR results are summarized in Figure S15. Peaks around 613 cm^–1^ are related to −CH2 rocking.^30^ The peak around 900 cm^–1^ is related to aromatic out-of-plane stretching of CH bonds. The pronounced peak at 3450 cm^–1^ corresponds to −OH hydroxyl group stretching. The band around 3200 cm^–1^ is related to the −NH group. The 2950 cm^–1^ peak is linked to −CH stretching vibration, and those around 1600 and 1700 cm^–1^ correspond to C=O and C=C bonds, respectively.^7^ The peak at 1100–1200 cm^–1^ represents the N–C O–C stretching in the molecules, which are also present in the FTIR spectrum for initial PD isomers.^31^

The FTIR spectra of the different CD compounds, obtained under different reaction parameters, show the presence of similar vibration bands, thus supporting the hypothesis that the CD structures are indistinguishable by other techniques and differ only in the carbonization direction on the molecular axis due to amine group location. The differences between all carbonized CDs are manifested mainly in the intensity variations in characteristic bands in FTIR spectra, supporting the hypothesis that all CDs have the same chemical groups with different percentages of bonds, which is supported by XPS data. The reason is the use of similar carbon sources that differ in carbonization directive growth due to constrained chemical paths, paved by the side groups of the growing molecules.^10^

#### 3.5.5 ^1^H NMR Nuclear Magnetic Resonance

To establish a more accurate placement of hydrogen in the molecular structure of CDs, ^1^H NMR spectra were measured from dried *m*-η8CDs. ^1^H NMR spectra (Figure S16) show an approximated ratio between hydrogens in the molecular structure. The hydrogens appearing at 1.1 ppm shift could be attributed to aliphatic hydrogens R–CH2–R, which can be related to some ring-opening of carbonized PD molecules that had left open ends or on some aliphatic −R–OH and −R–NH. The small peaks at 1.7 and 0.7 ppm are related to the more repeated R3–CH bond, which also occurred in a similar process or by EG addition. The shift at 3.1 ppm is correlated to bound R–OH and R–NH2 groups. The 3.3 ppm shift corresponds to the DIW trapped molecule peak. The 8 ppm shift corresponds to the aromatic −OH and −NH bonds, while the remaining peaks around 7–8 ppm belong to aromatic hydrogens Ar–H.

**Figure 5:**
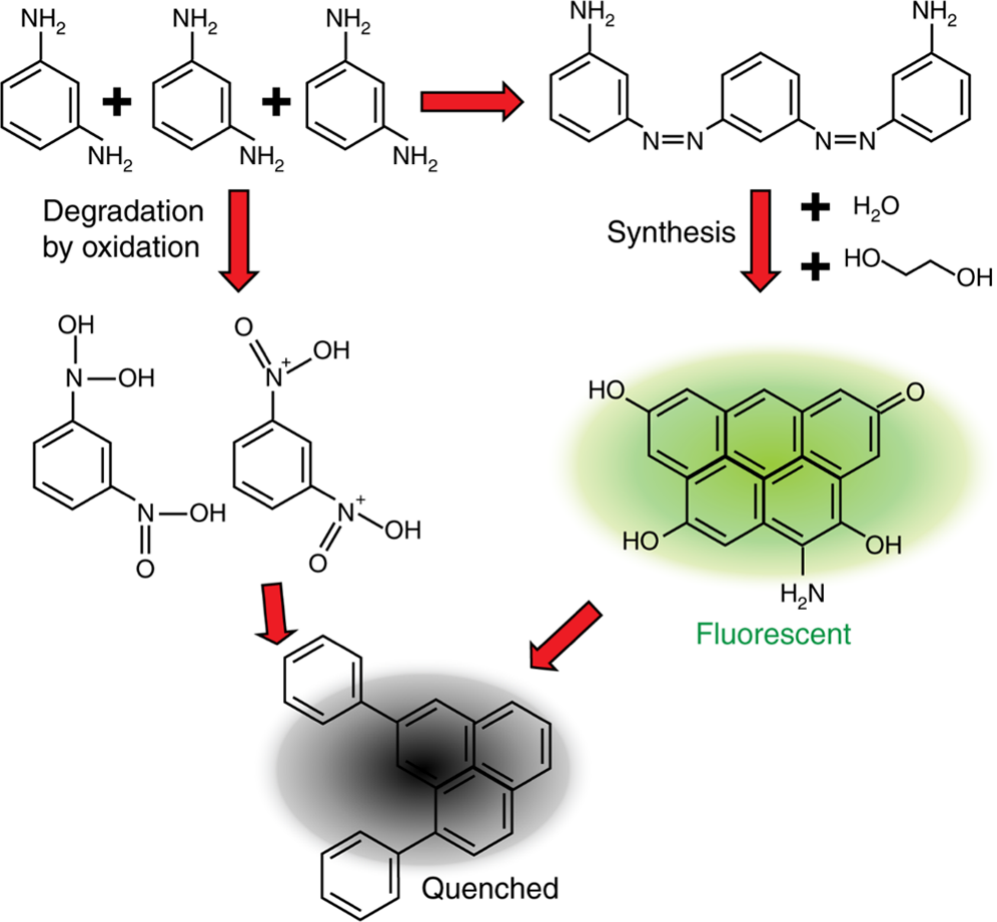
Proposed mechanisms of phenylenediamine carbonization through the pH-controlled hotspot model. The right path—nitrogen groups are involved, leading to the fluorescent carbon core with replacement of nitrogen by oxygen from water and ethylene glycol. The fluorescent core can also degrade into not a fluorescent structure. To the left degradation path that is caused by oxidized PD into nitro species, nitro groups are not active in the EG medium, resulting in the formation of inactive molecules.

A focus should be made on 3.6–3.8 ppm shifts, which can be attributed to the hydrogen atoms in ester carbonyl attached carbons R–CO–OCH2/–O–CH2. This type of hydrogen is the most abundant, which allows suggesting that the ring-opening/extension occurs at the more active spots of −NH2 at the side groups on the amino benzenes. Thus, amines play a crucial role in the carbonization process. A nucleophilic substitution of the amine with other fragments that are present during the reaction leads to a reaction hotspot for chemical growth (Figure 5). In contrast, the reaction fragments that form the final skeletal structure of the resulting fluorescent forms of carbon dots are ultimately controlled by the shielding protons at the reaction beginning. Consequently, the favorable product can be tuned through the initial pH, which in turn leads to the creation of molecular species that can carbonize into active forms.

From this data, we conclude several possible ratios: 5:5:3, 6:5:3, and 5:7:4 for aromatic, aliphatic, and hydroxyl/amine hydrogens, respectively. This will be further approximated to introduce our suggested structures.

#### 3.5.6 Structural Characterization Summary

From the data collected through the previous measurements, it is predicted that the final CD product size is averaged to 2–3 nm, with 350–700 a.m.u. FTIR data similarity in the apparent spectrum (Figure S15) suggests similarities in chemical properties of synthesized CDs. Moreover, the obtained FTIR spectra demonstrate similar bands, which can be observed by polymers of PD.^13^ Thus, we suggest that oligomerization of PD along with chemical changes affecting the side groups occurs during the carbonization reaction.

Based on atomic percentage and the O/N ratio of the CD composition, we hypothesize that the growth of CDs from PD monomers is initiated at the amine sites (Figure 5), resulting in the formation of low graphitic amines, which are further oxidized during synthesis. According to mass-spectrum data, the final molecular structure will probably contain four cyclic groups—two aromatics and another two semiconjugated pyridinic and pyrrolic rings with carbonyl and carboxyl on its periphery or in the rings meaning pyridinic and pyrrolic rings. These suggested structures will be briefly investigated in simulations.

#### 3.5.7 Growth Model and Molecular Dynamic Model

**Figure 6:**
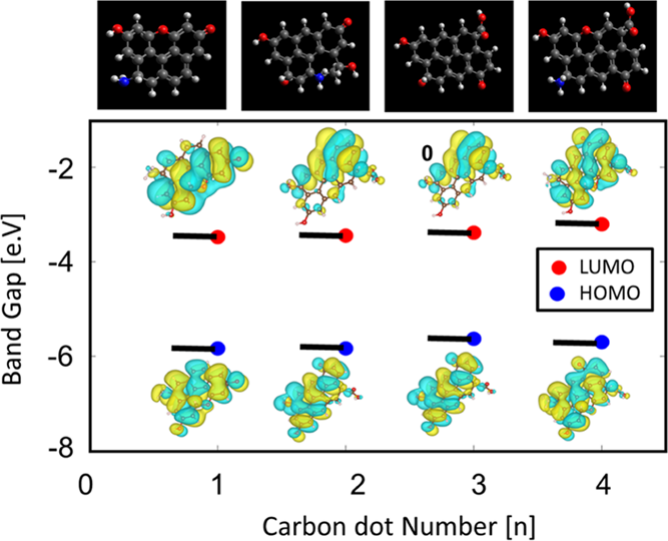
(A) Four suggested molecular structures for CDs (Avogadro software). Gray atoms are carbons, blue are nitrogens, red are oxygens, and white are hydrogens. (B) HOMO–LUMO energies calculated from DFT simulation with corresponding molecular orbital electron densities.

Simulations of some potential variants of CDs using the hybrid functional B3LYP were carried out to calculate the electronic band gap. According to the simulation results, the structures suggested here have a band gap between 2.2 and 2.4 eV (Figure 6), which can represent the first energetic state possible for radiative electronic relaxation. The suggested masses of structures are estimates of LCMS, where molecular fractions are about 300–700 a.m.u. The XPS data suggested a certain percentage of each atom, in particular, oxygen represents about 15–20% of the molecular mass. Relying on the proposed and supported by the XPS peak deconvolution, we suggest that the oxygen probably will be in a carbonyl or carboxyl form and bound to a ring, occupying pyridinic and furanic rings. All of the suggestions on the molecular structure of CDs based on structural characterization data allowed us to obtain a good agreement between molecular simulations and experimental spectroscopic data.^13^

An increase in the nitrogen fraction in suggested CD structures resulted in a lower band-gap value, which does not represent the characteristic energy gaps observed in PD dots, which was found to fall in the range of 2–3 eV (Table S5). The suggested molecules represent a simple case reaction that can occur under the condition of carbonization and thermal condensation. Additional data related to the atomic doping of the carbon shell and the carbon surface is found in the Supporting information.

### 3.6 Kinetic Model of Carbonization

A chemical reaction in a condensed phase is a stochastic process, with several paths, leading to different products. In our case, diversity of sizes, masses, and chemical compositions with time-dependence is expected to emerge. We assume that the carbonization reaction products can be divided into photoluminescent and nonphotoluminescent ones, which we take into account in our kinetic model. To regulate the complex kinetics of such a branched stochastic process, we introduce carbonization enhancers that simultaneously block other competing reaction paths.

According to the model, the desired product forms and degrades irreversibly. To quantify changes in the concentration, we are tracking the fluorescence emission intensity changes, which scale linearly with concentration, over the reaction time. Next, we change the main reaction environmental characteristics and observe the impact on RY and QY. The data of maximum intensity emission peak values were correlated to the concentration and fitted as
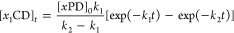
1where *k*_1_ is the carbonization rate, *k*_2_ is the degradation rate, *x*_0_ is the phenylenediamine isomer initial concentration, and *x*_1_ is the CD concentration. A more detailed discussion of the kinetic model can be found in the Supporting information.

Reaction dynamics output under different HCl concentrations (η and γ sets) was monitored by the fluorescence signal intensity. The samples were taken from a reaction media at given time intervals and measured using a plate reader, with the results summarized in Figure S17A and Figure S18. Reaction kinetics for different PD isomers was monitored by measuring the product fluorescence intensity. Data was fit to eq 1. for *po* mCDs reactions γ*m*, γ*p*, γ*o* (Figure S18A).

For all three isomers, the change in acid molarity resulted in major changes on the product emission properties and the reaction rates *k*_1_/*k*_2_. For the ortho isomer, only the set *o*-ηCDs displays its highest intensity with no acid (η1) with an emission peak at 570 nm. The acid addition shifted the peak after a certain pH threshold of 500 μL to 485 nm (Figure S10C). The set of *p*-ηCDs showed an increase in emission intensity that correlates linearly with an increase in acid molarity. A different dependency of emission intensity over reaction time was observed for *m*-ηCDs, with the highest fluorescence signal observed after 24 h of reaction and with 0.4 M yielding the highest intensity (Figure S17A). Validation of the anomalous behavior of *m*-ηCD carbonization was carried out by repeating the time series with a denser sampling rate (Figure S18B) for three different acid concentrations, which were marked as reactions η**7**m−η9m with 1.2, 0.6, and 0.12 M, respectively.

**Figure 7:**
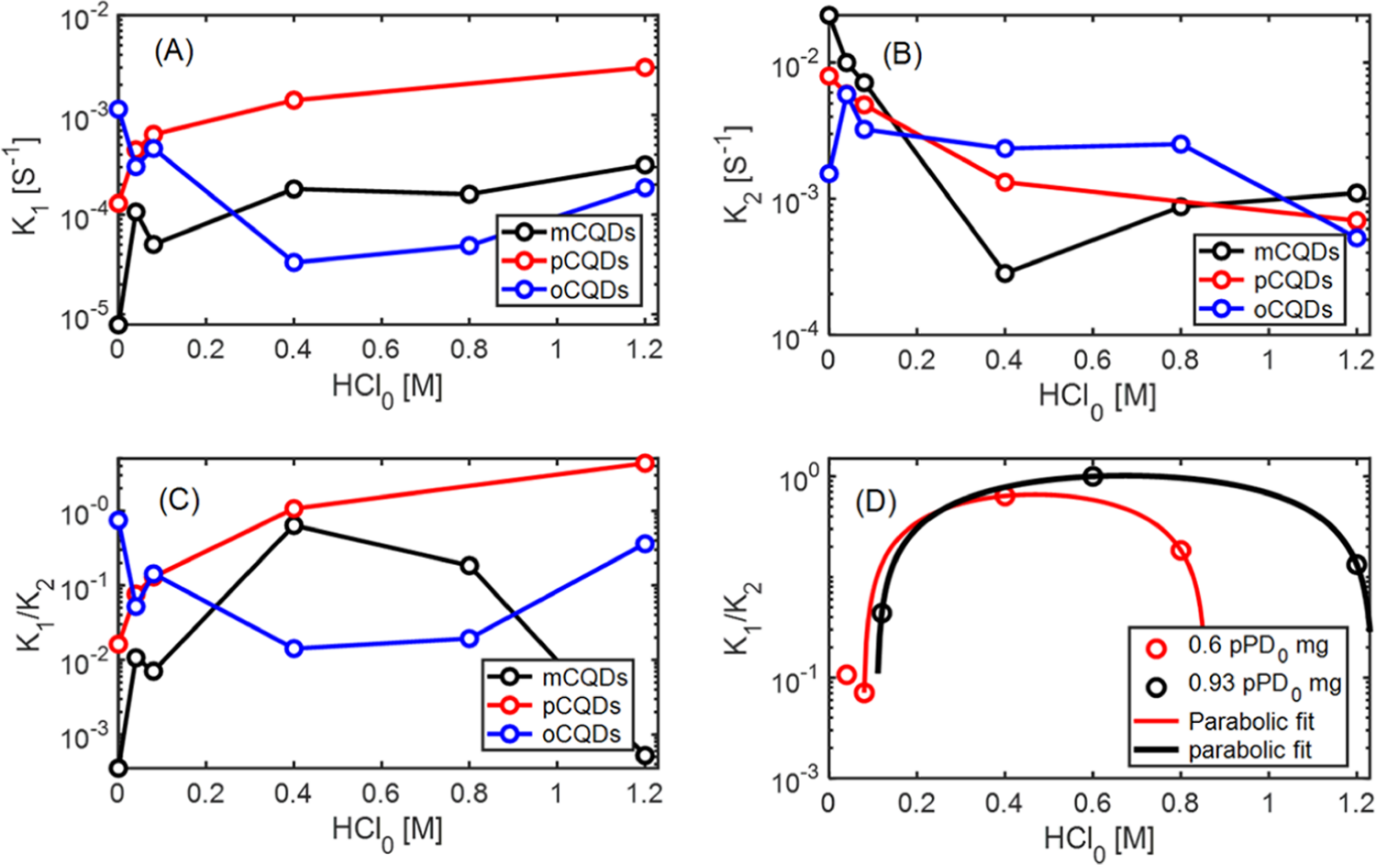
Reaction kinetics. *x*-Axes are the initial acid contents in molars, and *y*-axes are presented on a logarithmic scale. (A, B) Experimentally fitted rate constants of CD formation *k*_1_ and degradation *k*_2_, respectively, in first-order kinetics vs different acid molarities of reaction η1m−η6m (a–f) (black), η1o−η6o (a–f) (blue), and η1p−η6p (a–f) (red). (C) Ratio between formation and degradation *k*_1_/*k*_2_ vs the initial acid molarity η1−η6. (D) Ratio between formation and degradation *k*_1_/*k*_2_ vs the initial acid molarity of *m*CDs from η1m−η6m (black) and η7−η9m (red) plotted side by side with an emphasis on the optimal region that is fitted to the parabolic curve.

Reaction rate constants *k*_1_ and *k*_2_ were obtained by fitting experimental data of fluorescence change over time for different reaction bath acidities and are plotted in Figure 7A,B. One can notice that for *m*,*p*-η**-**CDs the *k*_1_ values curve increases with an increase in acid molarity, while *k*_2_ decreases (Figure 7A,B). Analysis of the data set for ηmCDs reveals a local minimum for the degradation rate. The rate constants change by an order of magnitude for the studied reaction bath acidities.

To expand our understanding of PD carbonization reaction dynamics and particularly *m*-ηCD anomaly, the value *k*_1_/*k*_2_ is employed as an estimation factor for optimal carbonization control in eq 1. The dependency of the generation/degradation constant ratio (*k*_1_/*k*_2_ factor) on reaction media acidity was used to analyze PD carbonization QY dynamics; dependency of *k*_1_/*k*_2_ values on acid molarity for all three isomers is shown in Figure 7C. *p*-ηCDs demonstrate a gradual increase, while *o*-ηCDs show two distinguishable maxima for high and low acidity values. *m*-η CDs show distinguishable acid molarity optimum for each reaction set (η1−η6/η7−η9), where *k*_1_/*k*_2_ reaches its maximum, which is also dependent on the initial *m*PD solution molarity (Figure 7D). The initial concentration of the PD isomer results in different optimal acid concentrations; 0.055 M *m*PD yields the highest *k*_1_/*k*_2_ value at an acid concentration of 0.425 M acid and 0.086 M *m*PD yields the highest *k*_1_/*k*_2_ value at 0.75 M.

However, the ratio  between protons and *m*PD molecules converges to ≈6.5 in both cases. Hence, this specific proton-to-*m*PD ratio (for *m*-ηCDs) allows minimization of degradation of *k*_2_ relative to the creation rate *k*_1_.

Another aspect to consider is the reaction rate since the usual interest is in a high product yield. CD solvothermal synthesis is a highly active reaction with a liquid solution at high temperature; the fluorescent products eventually degrade due to the reactive environment. The synthesized CDs transform into the nonfluorescent molecular form, which can be observed as an increase in solution absorbance with no visible fluorescence (energetic lossy molecules). Consequently, the degradation of CDs results in a decrease in QY of the product, which correlates with an increase in absorbance and a decrease in emission per the same number of molecules. Moreover, part of the initial CD precursors forms nonfluorescent molecular oligomers, which result in lower QY of the reaction product. All described processes further complicate the analysis of reaction kinetics. It is worth noting that some inactive products can be separated by centrifugation as mentioned in the Supporting information. Hence, achieving control over the kinetic parameters of a carbonization reaction provides an optimal product in terms of reaction yield, QY, and purification abilities.

According to the well-known collision theory, assuming that the diameter of molecules lies in the range of 2–5 nm and the mass range is 300–700 a.m.u at a constant pressure of 760 mmHg or 1 atm and temperature 424 K, the frequency of collision is calculated using , where *Z* is the frequency of collisions, *ν*_av_ is the average molecular velocity, and **λ** is the molecular mean free path. The minimal estimated frequency of collision value for 1 nm and 600 a.m.u yields *Z* ≈ 9.5 × 10^9^ [s^–1^], while the maximal value is calculated for a 5 nm particle with 400 a.m.u. and yields ∼2.3 × 10^11^ [s^–1^]. The estimated energy of activation in the Arrhenius model shows an average energy barrier between 110 and 125 kJ/mol. Activation energies are summarized in Table S7.

**Figure 8:**
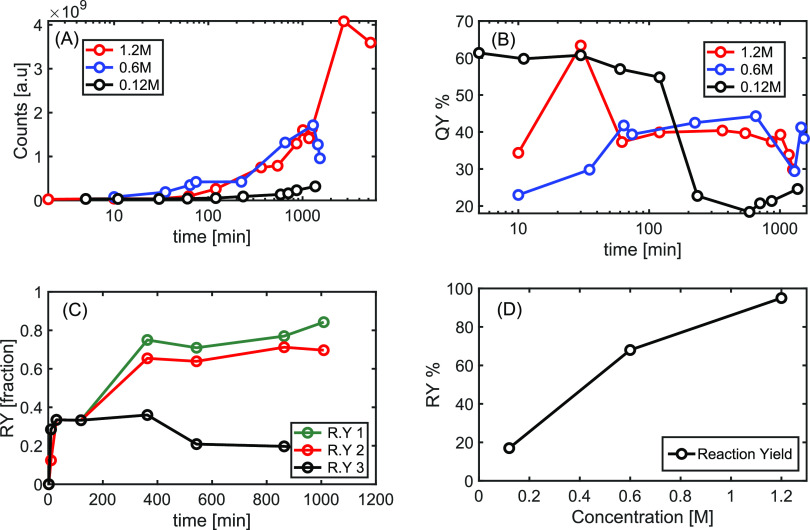
(A) *m*-η(7-9)CD emission peak as a function of time in a log scale. (B) QY as a function of time. (C) In terms of fraction from the mixture, RY1 corresponds to overall (green) fragments that maintain the same absorbance spectra, RY2 (red) corresponds to the brightest fractions only, and RY3 (black) is the abundance of the brightest fraction at 10.25 min. (D) RY as a function of the HCl initial molarity in the reaction.

Monitored fluorescence at different times shows significantly higher RY (Figure 8A); QY measurements showed that a relatively high acid concentration, above 50 mM, results in higher QY values (Table S4). The QY values’ dependency on reaction bath acidity is summarized in Figure 8B. The evolution of quantum yield during the reaction for a slow reaction or low acid molarity gives the best values during the first 3 h of the reaction time and allows one to reach QY values of >60% with RY around 20–30% (Figure 8A,D) owing to the low degradation rate. An increase in acid content up to 1.2 M allows one to achieve QY values of up to 45% for *m*PD after 2 h. However, with an increase in interaction time, the degradation of fluorescent CDs occurs, and the solution QY decreases.

The anomaly in *k*_1_/*k*_2_ maxima in *m*CDs, with relatively high creation to a low degradation rate, allows obtaining *m*CDs with QY of 42%, which drops after 1000 min at 453 K. Therefore, more photoactive products are generated, which compensates for the CD degradation that causes the QY drop (Figure 8B). The quantum yield values reach 60%, which is considered decent in a neutral water environment. Such high values of QY are among the highest for *m*PD carbonized products reported to date,^7,11,13,23−25,29,30,32^ paving its path into integration at photonic and nano-optical probing applications.

HPLC was used to establish reaction product composition dependency on the acid concentration. The mobile phase was acetonitrile/phosphoric acid at pH 2. Tracking the changes of *m*PD amounts in the reaction mixture, which aimed to solve numerically the full component kinetic mechanism, failed due to *m*PD instantaneous chemical decomposition when a strong acid is added to the initial reaction mixture. Figure S19 shows that the initial *m*PD is transformed to other forms, making its tracking a complex procedure. At an acid concentration of 0.6 M, some traces of *m*PD are observable in the chromatogram (Figure S19). Quantification of the consumed *m*PD during the first 3 h was 25–30% less than the initial value (Figure S19B,S20D). Optical probing of fractions detected at the chromatogram identified identical absorbance spectra that match that of the photoactive product spectra considered as our *m*-ηCDs (Figure S20C). The two main peak values were observed at 458 and 440 nm; all other substances are considered as side products with different spectra.

The RY1 (Figure 8C) is the total amount of photoactive compounds that are part of the total fractions that pass through the HPLC column, including fractions exhibiting inferior absorbance properties. The RY2 are the three main emitting fractions colored in the chromatogram (Figure S20A). The RY3 is the first fraction formed in the reaction with the absorbance peak at 460 nm, which is the closest to the emission band. The latter is the brightest fraction found in the mixture (Figure S20C, red curve). The calculated RY accounted for 5–10% of the separated inactive compounds during CD extraction. Moreover, the first fraction correlates with the highest QY (Figure S20) according to our estimations, which is consistent with higher QY values at the corresponding stages of carbonization (Figure 8B).

The chromatogram for *m*-η8 reaction products includes three main fractions corresponding to fluorescent products, yielding 20–30% RY. Estimation upon the relative area of the fraction is applied as calibration points for the area percentage of the same three active fractions in reaction *m*-η7, providing an estimated value between 70% RY for collection after 6 h. By comparing the optical absorbance and fluorescence intensity values at the emission peak, we observe consistency with HPLC estimation on RY by drying the extracted fraction: the same estimation on which after 2 h the product is 25–50% for reaction *m*-η**7**–*m*-η8 and a total of above 90% RY after several hours. It should be noted that the RY values included all contaminations presented as an inactive fraction by the HPLC diagram along with lower efficiency variants; yet, the obtained RY values are high compared to solvothermal methods reported,^11,33^ in particular PD carbonization.^34,35^Figure S20D summarizes the acid impact on *m*CD formation.

It appears that high acid content is related to sustained carbonization at longer times, making the reaction at its fastest rate, and preventing degradation forms. The impact on QY is not trivial as elaborated above.

### 3.7 Metal-Ion Enhancement/Quenching of Emission

**Figure 9:**
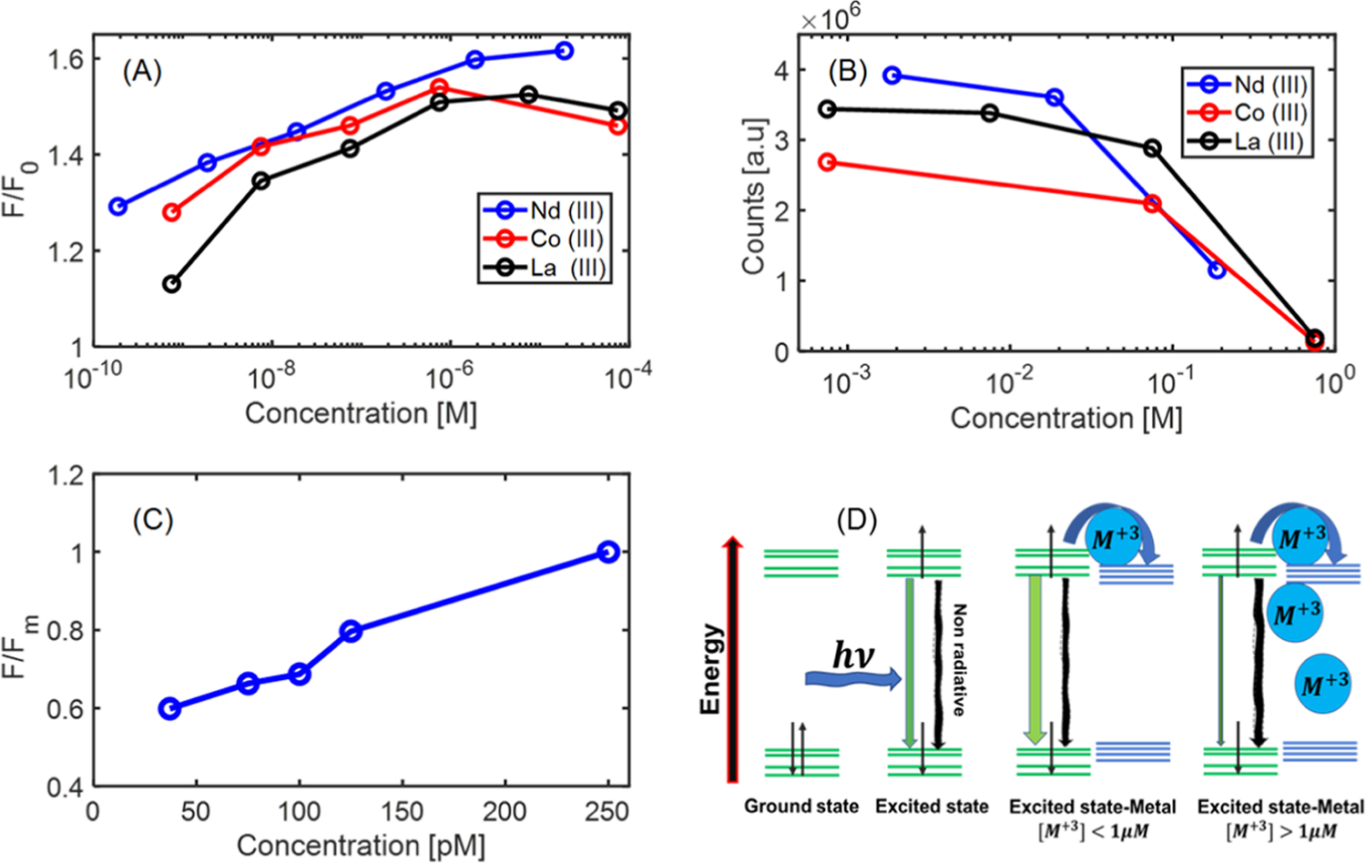
Metal-ion sensing. (A) Intensity enhancement. Metal-ion concentration at the range of 100 μM to 1 pM vs normalized intensity in the absence of metal ions in PBS buffer 0.0075 M; the scale is presented in a log scale. (B) Quenching regime at metal-ion concentration above μM. (C) Neodymium(III) enhancement at the picomolar range. (D) Proposed energetic path for the enhanced fluorescence by interaction of molecular orbitals with atomic d and f orbitals of the metal ion.

Metal-ion sensitivity is one of the popular properties of CDs.^2,3,5^ Side groups and chelating agents in the molecular structure of dots interact with metal ions through unpaired electrons. The ability of side groups to approach the positively charged metal ion is only one aspect of the interaction. The effect of metal ion on the electronic structure of the emitting molecules should be considered as well. To demonstrate metal sensitivity of the dots, we use Co(III), Nd(III), and La(III). The measurements were performed in DIW and in PBS buffer to normalize the changes occurring on emission due to charge balancing. Observation of fluorescence intensity vs metal concentration shows a pronounced response of the fluorescence enhancement due to the presence of metal ions. The enhancement was measured in PBS; thus, the effect is of chelating abilities that are directly linked to the electronic relevant energies related to the ground/excited states. Enhancement of emission when normalized to the absence of the metal reached 1.7× fold (Figure 9A).

Fluorescence response to the presence of the metal ion appeared in two distinct regimes, one is the enhancement regime and the other is the quenching regime (Figure 9B). The enhancement measured reached the detection limit of ∼pM. The sensing limit (10^2^–10^6^ pM) can be separated into subregions with a linear response. There is high responsivity to trivalent metal ions also in biological conditions. The high sensitivity to Co(III) can be useful for sensing this metal inside biological objects or for biological tests. The fluorescence enhancement observed in the presence of Nd(III) and La(III) heavy metal ions can be used for detection of subnanomolar concentrations of these elements. Another possible application of CDs is enhanced multi-bioimagers or OLED layers, which also have been demonstrated in previous works.^1−3^

On the other hand, a quenching region above 1 μM is found for metal ions, suggesting a wide band and high sensitivity in the enhanced emission at low concentrations. However, the quenching effect with linear regression appears above micromolar concentrations. The fluoresce of a single CD occurs due to excitation from a photon that places the electron in a higher energetic state. Singlet and triplet spin states can occur in the excited state. The presence of a metal ion at low concentrations enhances the emission by electronic interaction, which increases the rate of emitted electrons by electronic changes applied to CD states. At high concentration charge transfer for instance, or creation triplet CD species by the metal causing more irradiative processes to occur (Figure 9D). The multitasking multicolor nanodots demonstrate high-performance properties in terms of QY, tunability, and great sensing abilities, thus paving their path into further applications in the future.

## 4 Summary

In this work, a new method for phenylenediamine carbonization was developed. EG served as the global medium for carbonization. Under high viscosity and mild heating, a controllable process was carried out through a semiclosed refluxed system. Variation of chemical bath acidity allowed one to control the carbonization reaction, altering a particular growth path for *m*CDs. High acidity above 0.6 M results in a high RY of up to 90% and QY above 60%. Addition of hydrochloric acid into the reaction bath at the concentration of >0.1 M allowed suppressing the fluorescent CD quenching/decomposition. Molecular mechanism and molecular dynamics were applied to support the results. Kinetic analysis shows a general increase in CD formation rates of ∼10-fold, while there is a significant drop in the degradation rates of ∼100-fold compared to the neutral environment. Optimizing the synthesis parameters allowed tailoring the optical properties of the PD CDs, yielding on-demand colorful dots. Structural characterization of resulting CDs allowed us to assume oligomerization as the most plausible way of CD formation under given reaction conditions. Relying on CD characterization, simulations were employed to link the molecular structure with electronic band gaps. The emitting CD variants are directly related to the chemical nature of the randomly occurring CD. Moreover, high responsivity to metal ions down to 50 pM places this fluorescent probe in the high-sensitivity zone.

The colorful spectral tuning along with metal responsivity allows further integration of PD CDs into a wide range of applications-driven research.

## Supporting Information Available

Materials, details of synthesis parameters and extraction, PLE spectra, lifetime decay statistics, TEM, LCMS spectra evolution in time, XPS, FTIR, molecular simulations of CDs, kinetic modeling equations, HPLC, and QY measurement details (PDF)
