# Supplementary material for: Multicolor Phenylenediamine Carbon Dots for Metal-Ion Detection with Picomolar Sensitivity
Source: ACS Appl Nano Mater. 2021 Sep 8;4(9):9919–31. doi: 10.1021/acsanm.1c02496 (PMC8488935; doi:10.1021/acsanm.1c02496)
Supplement: Supplementary file 1 — an1c02496_si_001.pdf [file an1c02496_si_001.pdf]

Multicolor Phenylenediamine Carbon Dots for Metal Ion Detection with Picomolar Sensitivity

Hani Barhum[1,2,*,=], Tmiron Alon[1,2,=], Mohammed Attrash[3], Andrey Machnev[1,2], Ivan Shishkin, and Pavel Ginzburg[1,2,4]

[1]Department of, Ramat Aviv, Tel Aviv 69978, Israel

[2]Light-Matter Interaction Centre, Tel Aviv University, Tel Aviv, 69978, Israel

[3]Schulich Faculty of Chemistry, Technion – Israel Institute of Technology, Haifa 32000, Israel

[4]Center for Photonics and 2D Materials, Moscow Institute of Physics and Technology, Dolgoprudny, 141700 Russia

Supplementary information

1. Materials

Chemicals were purchased by Merck group and were used without any further purification. Meta-phenylenediamine (mPD) >99%, ortho-phenylenediamine (oPD)>99%, para-phenylenediamine (pPD)>99% Ethylene glycol (EG)>98%, Poly Ethylene glycol 200/400/600 99>%, Propylene glycol (PG)>99% Dimethylformamide >99.9&, Ethanol A.R 100%, Concentrated acids: (70%) Nitric acid , Sulfuric acid 99%, Hydrochloric acid 32% , phosphoric acid 85%, Acetic acid 99%, Dimethyl Sulphoxide AR, Chloroform AR, Methylene Chloride AR, Tetrahydrofuran AR, fluorescein 95%,$CoCl_3$ 99%,$NdCl_3$ 99.99%, $LaCl_3$ 99.9% MilliQ water 18MΩ.

Phenylenediamine isomers (meta-(mPD) ortho-(oPD) and para- (pPD)) for carbon dot synthesis were purchased from Sigma Aldrich and used with no additional purification. EG, PEG 200/400/600/ PG purchased from Sigma Aldrich were used as reaction medium. Hydrochloric, Sulfuric, Nitric, Phosphoric, and Acetic acids were used for controlled change in the acidity of reaction bath.

1.2 Purification of CDs

The reaction mixture was mixed with acetonitrile 1:10 v/v then vortexed for 1min, followed by 5 min centrifugation at 15k rpm. Precipitate washed with anhydrous ethanol to collect any remaining fluorescent CDs. The participation can be dissolved in DIW. The precipitate was tested for fluorescence activity, also the fluorescence of purified and unpurified CDs was compared. The acetonitrile

solution was treated more for further extraction, carbon dots solution is dried at the chemical hood. Drying may be accelerated by mild heating to ensure that there is no significant thermal degradation. Then the remaining solution is transferred to vacuum chamber for 48h. Each reaction volume was monitored at each time point of collection. The missing volume was mainly evaporation at first 24hours.

2. Reaction medium impact on fluorescence properties of CDs

Photoluminescence (PL), photoluminescence excitation spectroscopy (PLE), and absorbance spectra were measured with a plate reader Synergy H1. The working spectral range of the H1 is 300-700nm for excitation and emission, while 230-900nm band, sampled with 1 nm step, is available for absorption measurements. Horiba Jobin Yvon FL3-11 spectrofluorometer was used for Fluorescence measurments. Lifetime measurements were done with a PicoQuant system, which uses Taiko picosecond diodes as a 375 nm excitation. The collected light was split by 50:50 beam splitter to CMOS camera (Thorlabs DCC1240C) for imaging and fiber-coupled Andor Kymera 193i spectrometer equipped with iDus 401 TEC-cooled CCD) for Spectrum analysis, and PicoQuant PDM photon counter for lifetime measurements.

Reaction types are summarized in Table S1.

Reaction Symbol	Solvent medium for carbonization	Additives to the medium and their percent	Variations/Products	Temperatur e
α	EG 15mL	-------------------------	meta(m-α),<br>para(p-α),<br>ortho (o-α)	453K
β	EG 15mL	DIW β1<br>HCl 1M:<br>500uL β2<br>100uL β3<br>- KOH 1M :<br>100uL β4<br>500uL β5	meta(m-β),<br>para(p-β),<br>ortho (o-β)	453K (βa),<br>513K (βb),<br>573K (βc)
γ	EG 50mL	HCl 12M<br>10mL	meta(m-γ), para(p-γ),ortho(o-γ)	

δ	EG 15mL	10% Concentrated acids 1.5mL	δ1(Hydrochloric),<br>δ2(Sulfuric),<br>δ3(Phosphoric),<br>δ4(Nitric),<br>δ5(Acetic))	
ε	ε1(Propylene Glycol),<br>ε2(Glycerol),<br>ε3(Dimethylformamide),<br>ε4(EG)<br>15mL	10% Hydrochloric acid 12M<br>1.5mL	Para, meta	
ζ	PEG-200(ζ1), PEG-400(ζ2), PEG-600(ζ3), EG (ζ4) 15mL	10% HCl 12M<br>1.5mL	para, meta, ortho	
η	EG 15mL- and 100mL	0HCl(η1), 50uL HCl(η2), 100uL HCl(η3), 500uL HCl(η4), 1000uL HCl(η5), 1500uL HCl(η6),<br>1%HCl (η7) 1mL, 5%HCl (η8) 5mL, 10% HCl(η9) 10mL	η1-6 ortho, para, meta<br>η7-9 meta	

Table S1. Reactions- experimental details.

2.1 Reaction evolution in neutral medium meta(α1)/para(α2)/ortho(α3) solutions (10mg/mL) were prepared by dissolving in ethylene glycol and heating up on hot plate at 433K for various times. Samples over reaction time were taken when visible changes in the solution color could be observed. Spectral evolution of ***α***-reaction sets under different excitations is shown in Fig.S1.

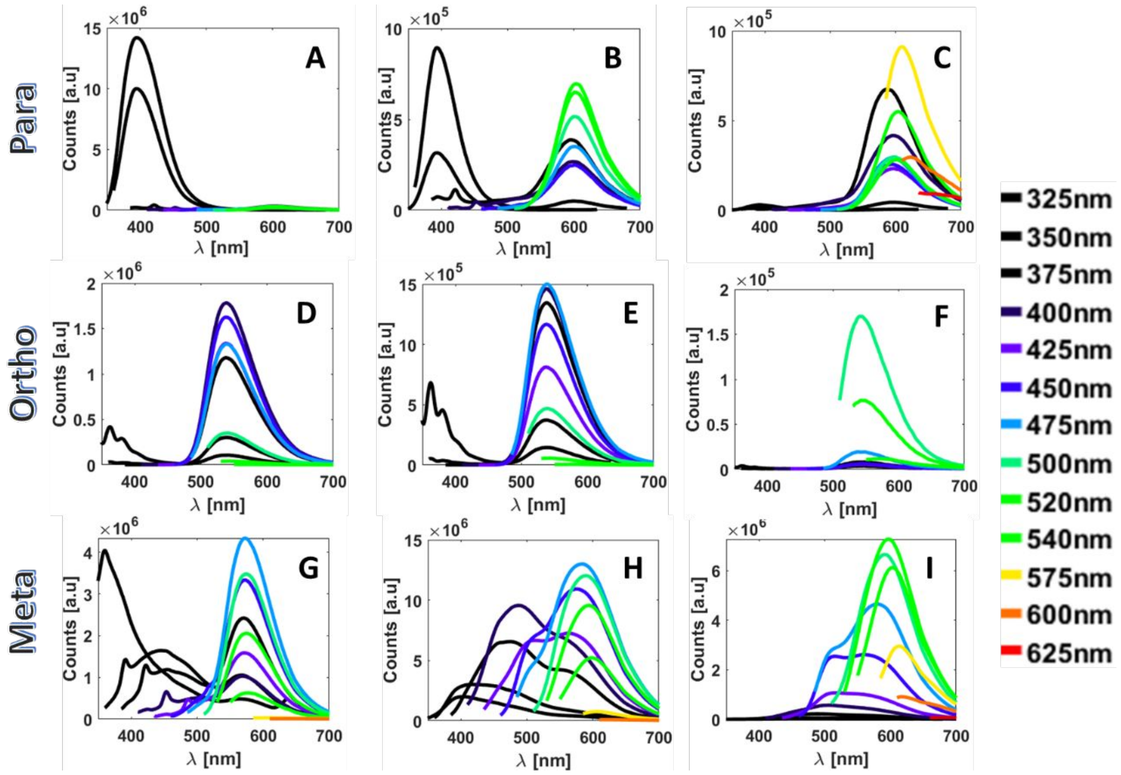

Figure S1. (A,B,C) p-αCDs (D,E,F) o-αCDs. (G,H,I) m-αCDs. Spectra were collected at different sample extraction times -- (A,D,G) 15 mins (B,E,F) 60min (C,F,I) 135min the colors of the spectra related to the excitation color.

2.2. Reaction medium acidity impact

2.2.1. pH impact on CDs fluorescence

Symbol	Isomer	Additives	Medium pH
β1	meta (m - β1)<br>para (p - β1)<br>ortho (o - β1)	Only DIW	6
β2	meta (m – β2)<br>para (p – β2)<br>ortho (o – β2)	100uL HCl	0.2
β3	meta (m – β2)<br>para (p – β2)<br>ortho (o – β2)	500 uL HCl	1.3
β4	meta (m – β2)<br>para (p – β2)<br>ortho (o – β2)	100uL KOH	12.7
β5	meta (m – β2)<br>para (p – β2)<br>ortho (o – β2)	500uL KOH	13.8

Table S2. Reaction medium acidity modification additives

10mg of PD isomer was dissolved in 10:1 EG-water mixture. Medium pH was controlled by adding acid (HCl) or base (KOH) solution. The reactions are listed in Table S2. Temperatures of the reaction bath was varied to three different cases: 453K, 513K and 573K.

Excitation was scanned in range between 325 to 600 nm in 25nm steps.

pH-dependent emission spectra under different excitation wavelengths for p- o- m- p- CDs under different reaction acidities and temperatures are presented in Figs. S2-S4 respectively.

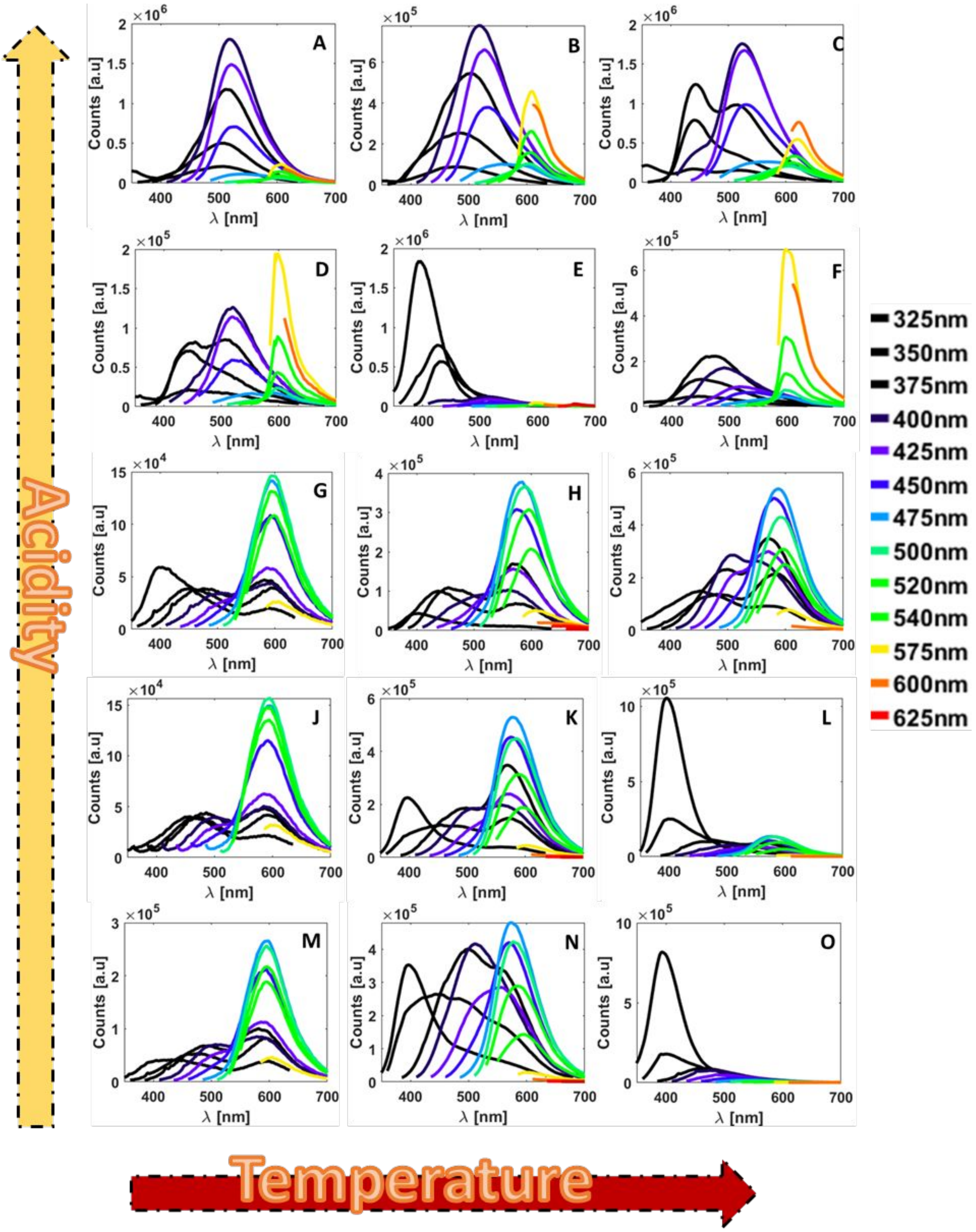

Figure S2. Excitation-dependent fluorescence of p-βCDs under different acidities and synthesis temperatures, spectra colors represents the excitation wavelength.

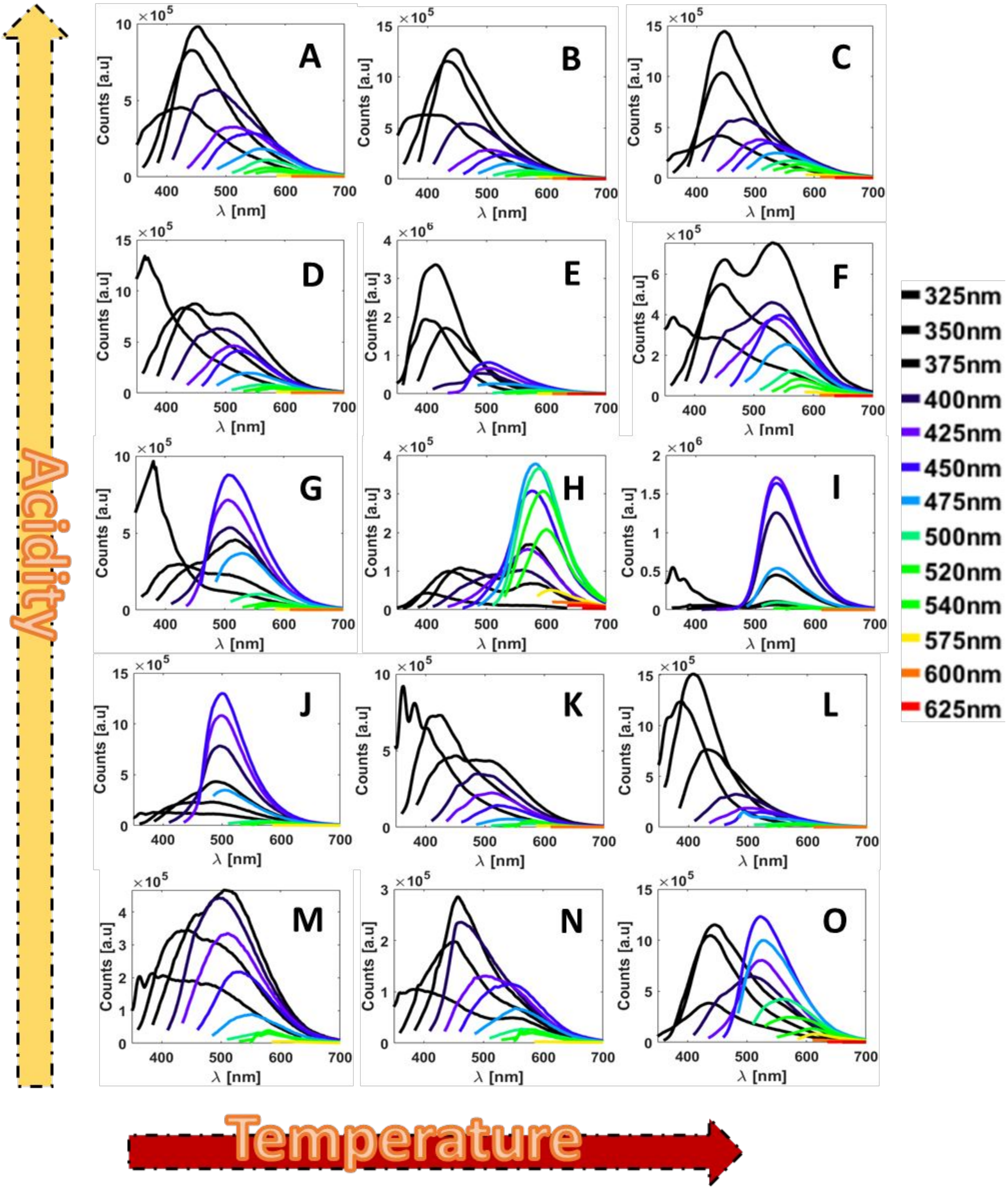

***Figure S3.** Excitation-dependent fluorescence of o-βCDs under different acidities and synthesis temperatures, spectra colors represent the excitation wavelength.*

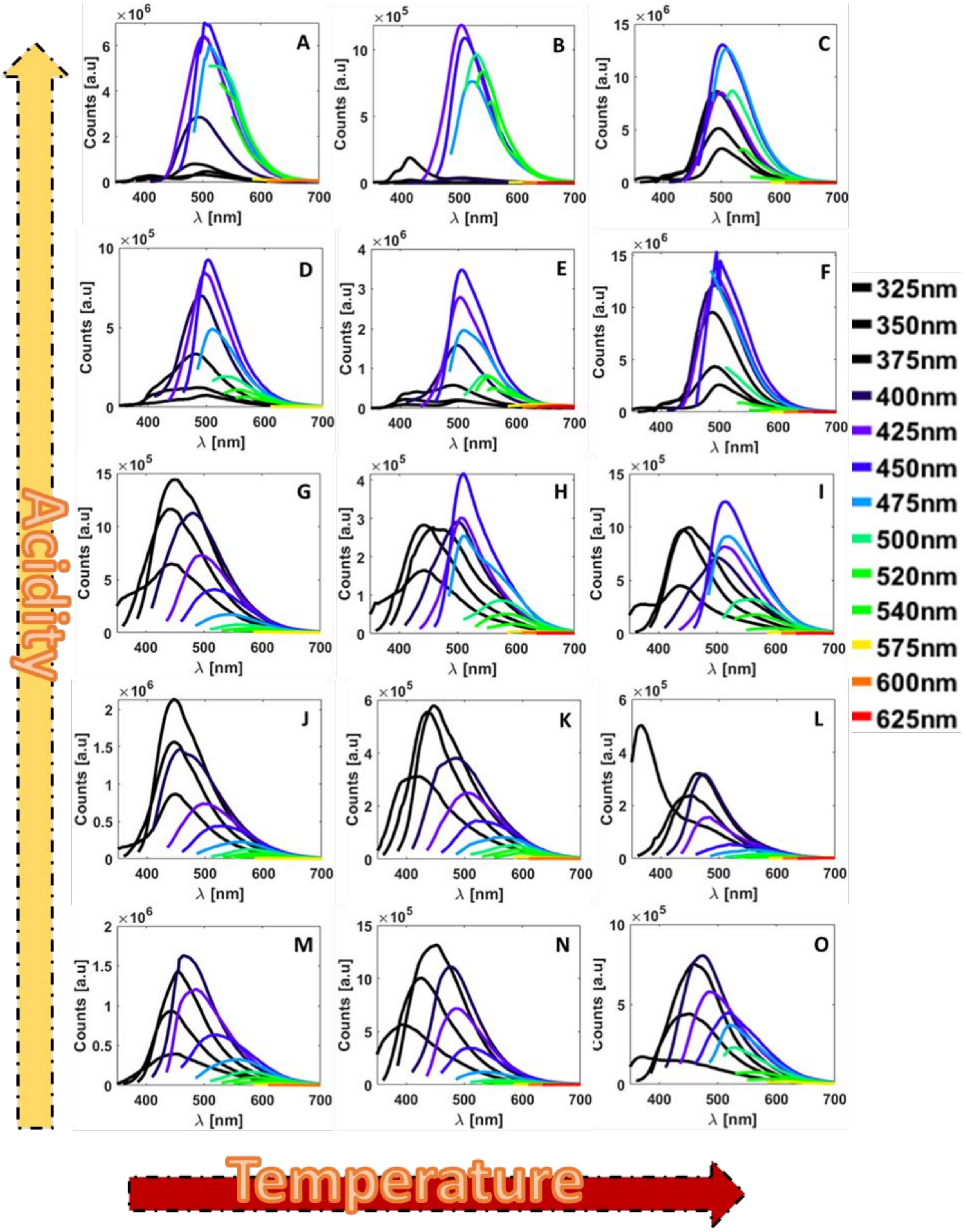

Figure S4. Excitation-dependent fluorescence of m-βCDs under different acidities and synthesis temperatures, spectra colors represent the excitation wavelength.

2.3.3 Fluorescence evolution in presence of high acid contents

CDs formation output was monitored by taking fluorescence spectra (420 nm excitation) of samples collected at different times after reaction was initiated. Reaction was carried in 250mL three-necked flask with attached refluxed system. 0.5mg PD was dissolved in 50mL EG with 10 mL concentrated 12M hydrochloric acid. Reaction temperature was set to 433K. Carbon do ts obtained under these conditions are referred as γm-CDs, γp-CDs, and γo-CDs respectively. Results are summarized in Fig. S5.

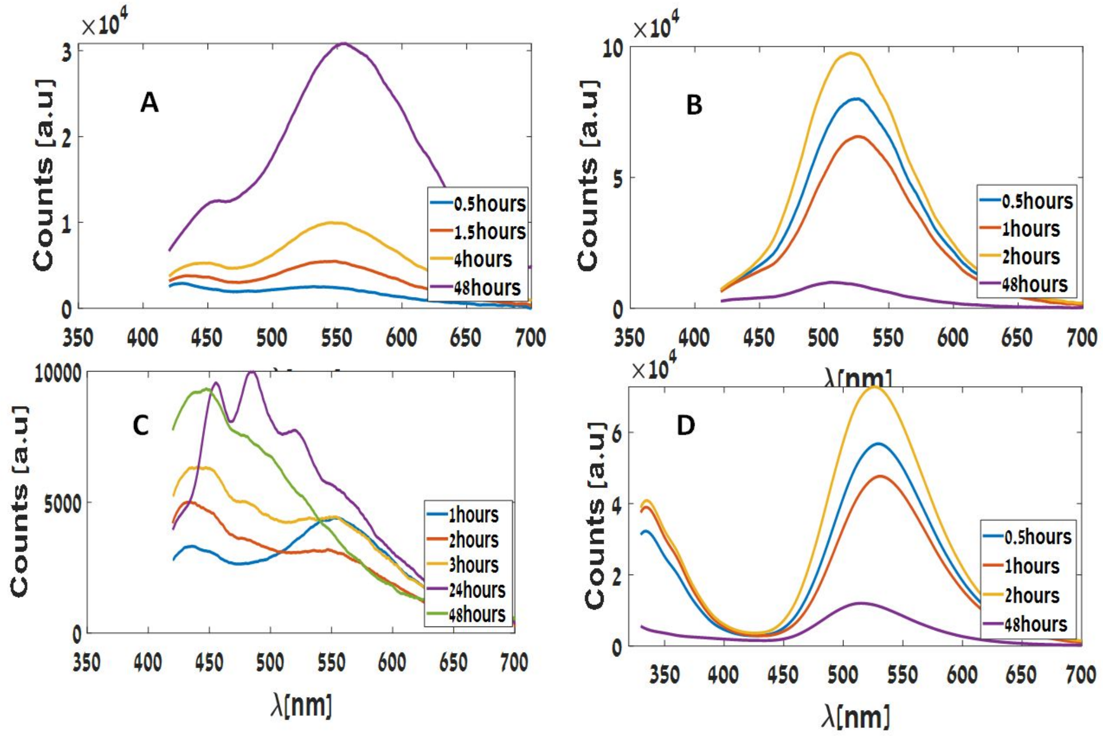

Figure S5. Emission spectra under 420nm excitation of γ-CDs prepared in 12M Hydrochloric acid collected at different times from reaction beginning in a total interval of 48 hours. A. p-γCDs B. m-γCDs C. o-γCDs D. Emission of m-γCDs pumped at 300nm.

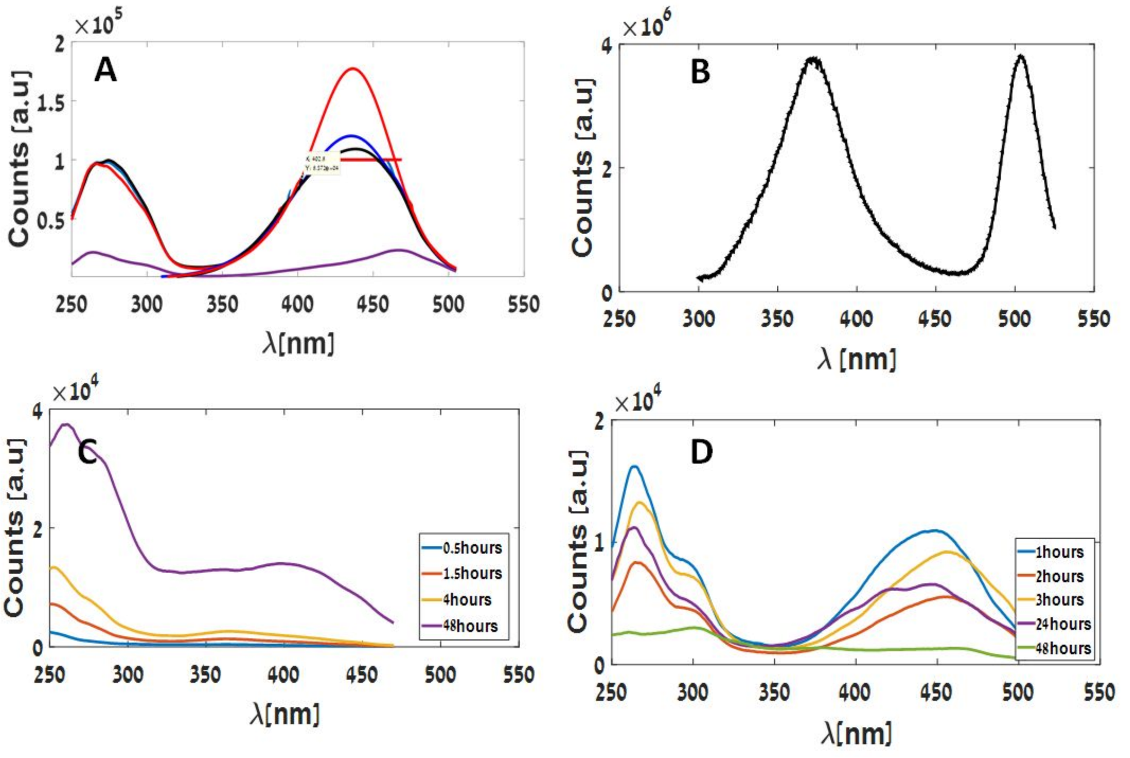

Figure S6. PLE spectra of γ-CDs at different emission wavelengths (A) o-γ*CDs collected after 24 hours PLE at different times (B) m-γCDs collected after 48 hours Emission intensity at 528nm with excitation sweep (C) p-γCDs collected after 6 hours PLE at 500nm and (D) p-γCDs collected at different reaction times, PLE spectra at 554nm.*

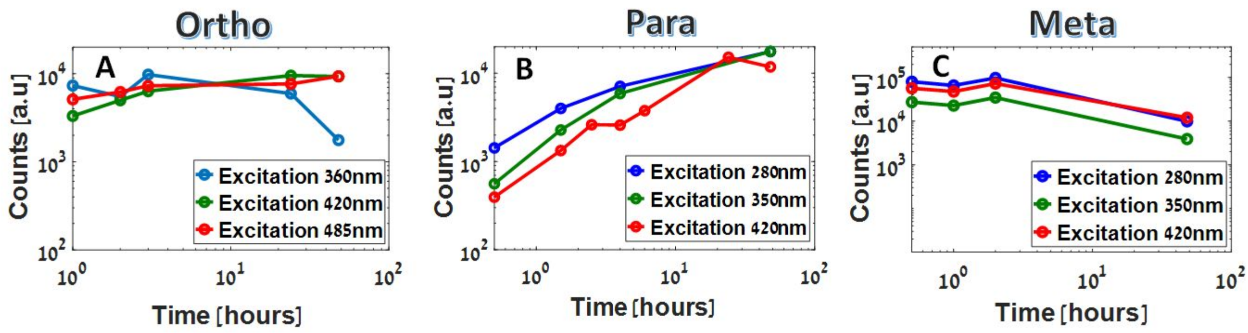

Figure S7. (A) γ-oCDs (B) γ-pCDs (C) γ-mCDs emission intensity vs. time for different excitation wavelengths.

2.3.4 Acid and solvent impact

Reaction mixtures were prepared in 20mL scintillation vials by adding 10% (1.5mL) concentrated acids to total volume of 15ml (6.6mg/mL) mPD solution. Carbon dots from these experiments will be referred to as δ1(Hydrochloric 12M), δ2(Sulfuric 18.6M), δ3(Phosphoric 14.8M), δ4(Nitric 15.5M), δ5(Acetic 17.6M).

The best acid was chosen in terms of QY for testing different solvents. We chose hydrochloric acid as 10% of aqueous content with 90% different solvents. ε1(Propylene Glycol), ε2(Glycerol), ε3(Dimethylformamide), ε4(Ethylene Glycol).

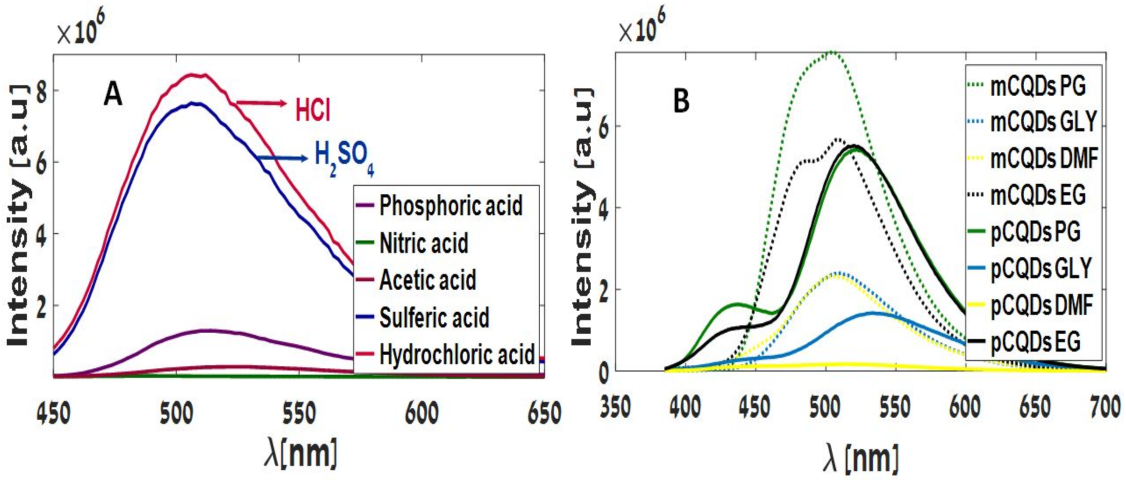

Figure S8. A. Emission spectra of mCDs δ set at 420nm excitation. All the samples were prepared in concentrated acids. The highest intensities marked in blue and red, which are sulfuric and hydrochloric acid respectively B. Emission spectra of mCDs ε **set** ***(dashed) and pCDs (Solid), in a different organic solvent, excited at 375nm.***

Various solvents interact differently with acids and PD molecules. The affinity between reaction components, global steric factors of Solvent-EG, and protonation dynamics are all a matter of solvent. Emission characteristics and intensities in the set ε1-ε4, for both for mPD and pPD, are recorded (Fig S8B). Generally, both ε4 m/p CDs attained the highest emission properties RY-QY. The ε1 environment of propylene glycol showed similar results (Fig S8B), as predicted, due a physicochemical similarity with EG. Spectral emission and intensity are almost identical for ε1, ε2, and different for ε3. Supporting the assumption on chemical environment similarity. To avoid decoherence in the work, and for consistency, we stick to the higher QY results of dots prepared in

EG. Further investigation concerning the solvent nature is yet to be established. Thus, this could be a question to be addressed in a future research.

2.3.5. Viscosity impact on CD carbonization

Viscosity changes were obtained by using identical chemical affinity solvents to the reacting molecules thus PEG 200(ζ1)-PEG 400(ζ2)- PEG 600(ζ3), EG (ζ4) gave the desired conditions. The reaction was held in 1.2M HCl concentration and a total volume of 15mL.

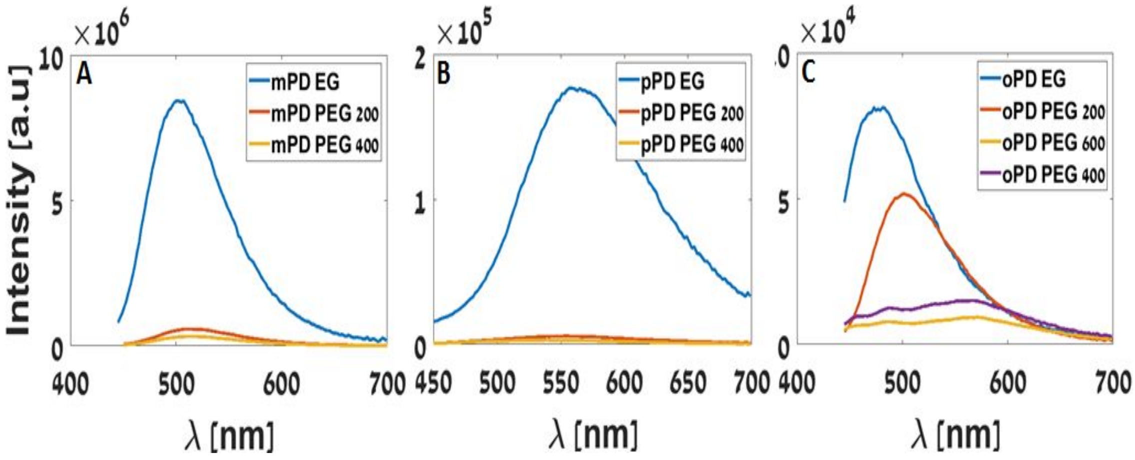

Figure S9. Phenylenediamine CDs emission spectra at 420nm excitation pump. Each group reacts differently to the poly EG solvents. Each spectrum represents different preparation viscosity with 1.2M acid in 10% DIW 90% EG A. m-ζCDs B. o-ζCDs C. p-ζCDs

2.3.6 Synthesis in different acid content:

100mg of PD isomers was placed in 20mL vials. The total reaction volume was set to 15mL, 13.5mL EG, and 1.5 aqueous solutions with 0 (η1) – 50 (η2) – 100 (η3) - 500 (δ4) – 1000 (η5) – 1500 (η6) uL concentrated HCl. Samples extracted at 5 points.

mPD reaction was done also in a refluxed system at 250mL at round three-necked flask with temperature adjusted to 150 ^{0}C. 1g of mPD in 100mL reaction volume, 90mL EG and 10mL aqueous HCl to gain the initial concentration of [0.12M (η7) -0.6M (η8) -1.2M (η9).

2.3.7 CD emission lifetimes

CDs emission lifetimes were measured using a home built TCSPC setup around SPAD detector (MPD photonics) coupled to spectrograph (Andor Kymera 193) with 375nm picosecond pulsed laser (PicoQuant) used for excitation. Reaction set η was used for CDs emission analysis. The η-mCDs have emission bands around 520nm. ηoCDs peak appears around 485nm with two bands at 450 and 530nm respectively (Fig S9a-c). ηpCDs contribute to a broad emission from 530nm to 600nm with apparently two overlapping peaks (Fig S9b).

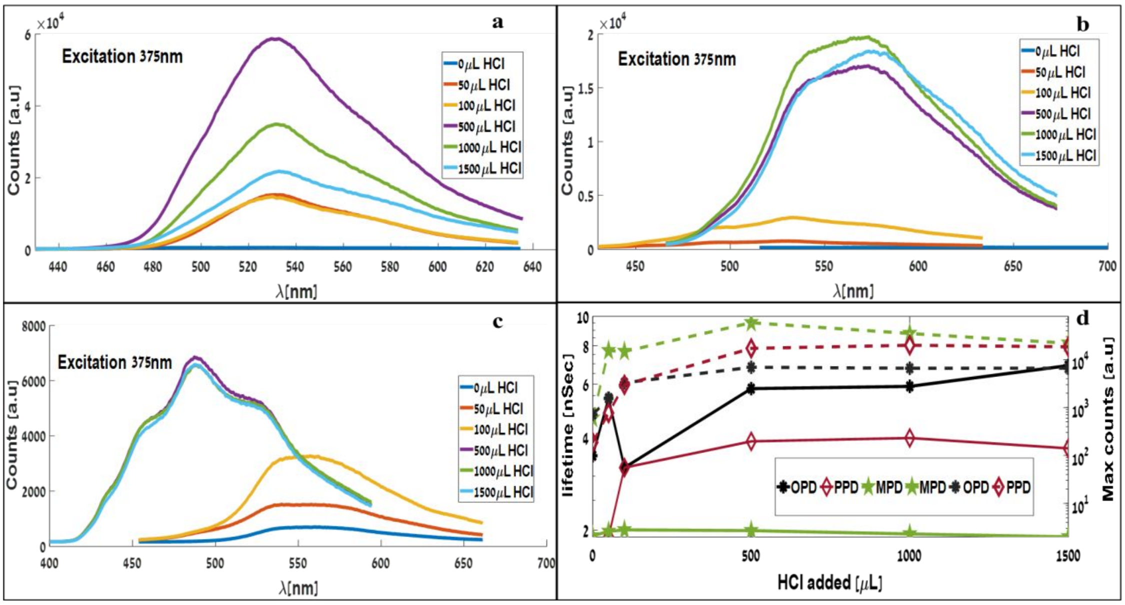

Figure S10. Emission Spectra of PD CDs when pumped with 375nm picosecond laser a. η***mCDs from reactions*** $m\eta\ (1-6)$ ***b. p-ηCDs from reactions*** $p-\eta\ (1-6)$ ***c. o-ηCDs from reactions*** $o\eta\ (1-6)$ ***d. lifetime mean value of the corresponding CDs and its maxima***

dynamics solid line, max intensity counts value for the corresponding ηx dashed lines.

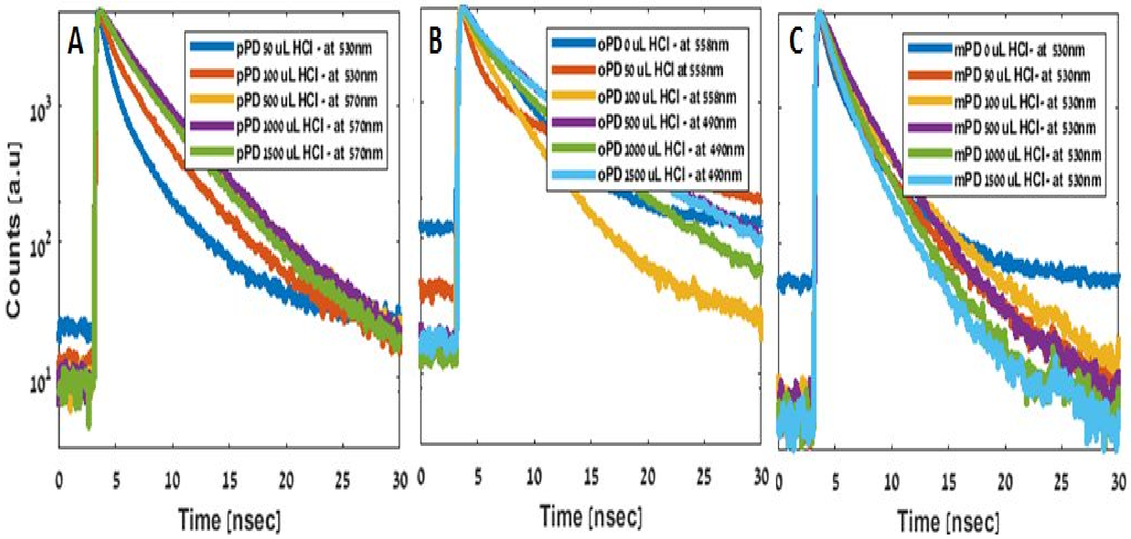

Figure S11. Lifetime decay of ηCDs series. a. p-η(1-6)CDs b. o-η(1-6)CDs c. p-η(1-6)CDs The count was stopped at 5000phtons detected at the photon counter for all measurements. The time scale of decay was typical for fluorescent dye with characteristic decay in the range of 2-7ns with two main competitive radiative relaxation processes.

3. Structural characterization

. In order to understand the changes addressed to phenylenediamine molecules during carbonization, we have done FTIR, mass spectra and XPS analyses. We believe, all of these will enable to introduce a hypothetical model for CDs formation.

3.1 Transmission electron microscopy (TEM)

Transmission electron microscopy (TEM) images were collected with a Jeol JEM 1011 (Jeol, Japan) electron microscope operating at an acceleration voltage of 100 kV and recorded with an 11 Mp fiber optical charge-coupled device (CCD) camera (GatanOrius SC-1000). For the sample preparation, 1 μL of the diluted sample was dropped onto a carbon-coated copper grid, and the solvent was removed by evaporation at room temperature.

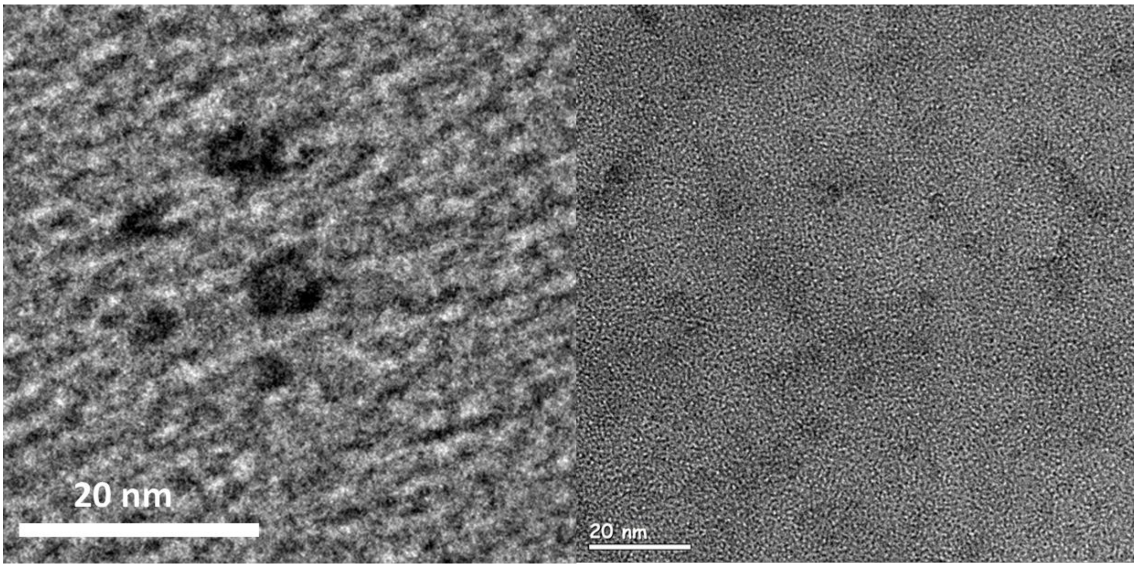

Figure S12. TEM image of extracted CDs of the reaction m-η4 the diameter of the particles is 2-4nm (Right) Individual particle captured (Left)

3.2 Dynamic light scattering (DLS) and Zeta potential measurements

The size distribution of particles and their surface zeta potential was measured in DLS mode with Malvern Zetasizer Nano Series running DTS software.A He-Ne laser source operates at 633 nm with maximum otput power of 4 mW.. Analysis performed at an angle of 173 and a temperature of 25C. Since CD is a small molecule with a low scattering cross-section at the laser wavelength, the measurement setup detection limit is 0.1mg/mL all the samples were done in an aqueous solution at 1-2mg/mL concentration.

3.3. Liquid chromatography Mass Spectra

The mass-spectra of each reaction product was taken after different reaction times. Using the C18 column to charge and separate the phases. The peak at 107-109 a.m.u. corresponds to the initial unreacted product (phenylene diamine isomer). The peak intensity appears in percent of the fraction mass from other similar fractions and not from the total mass distribution

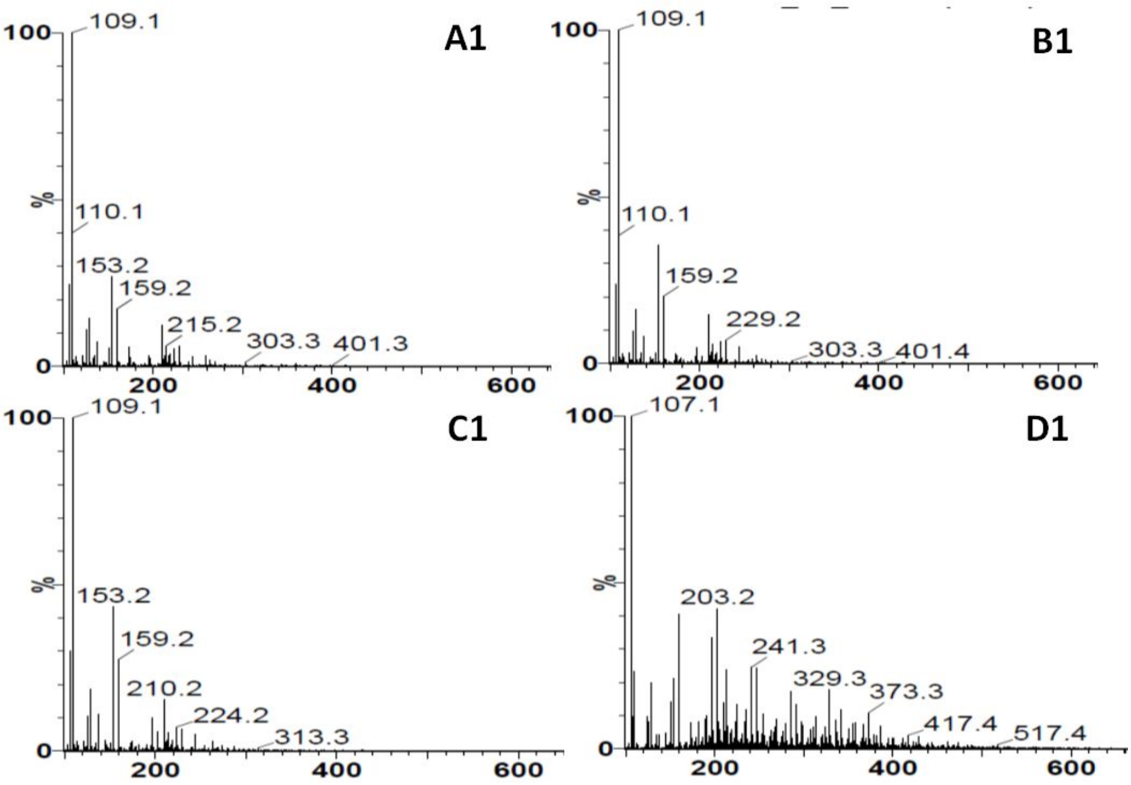

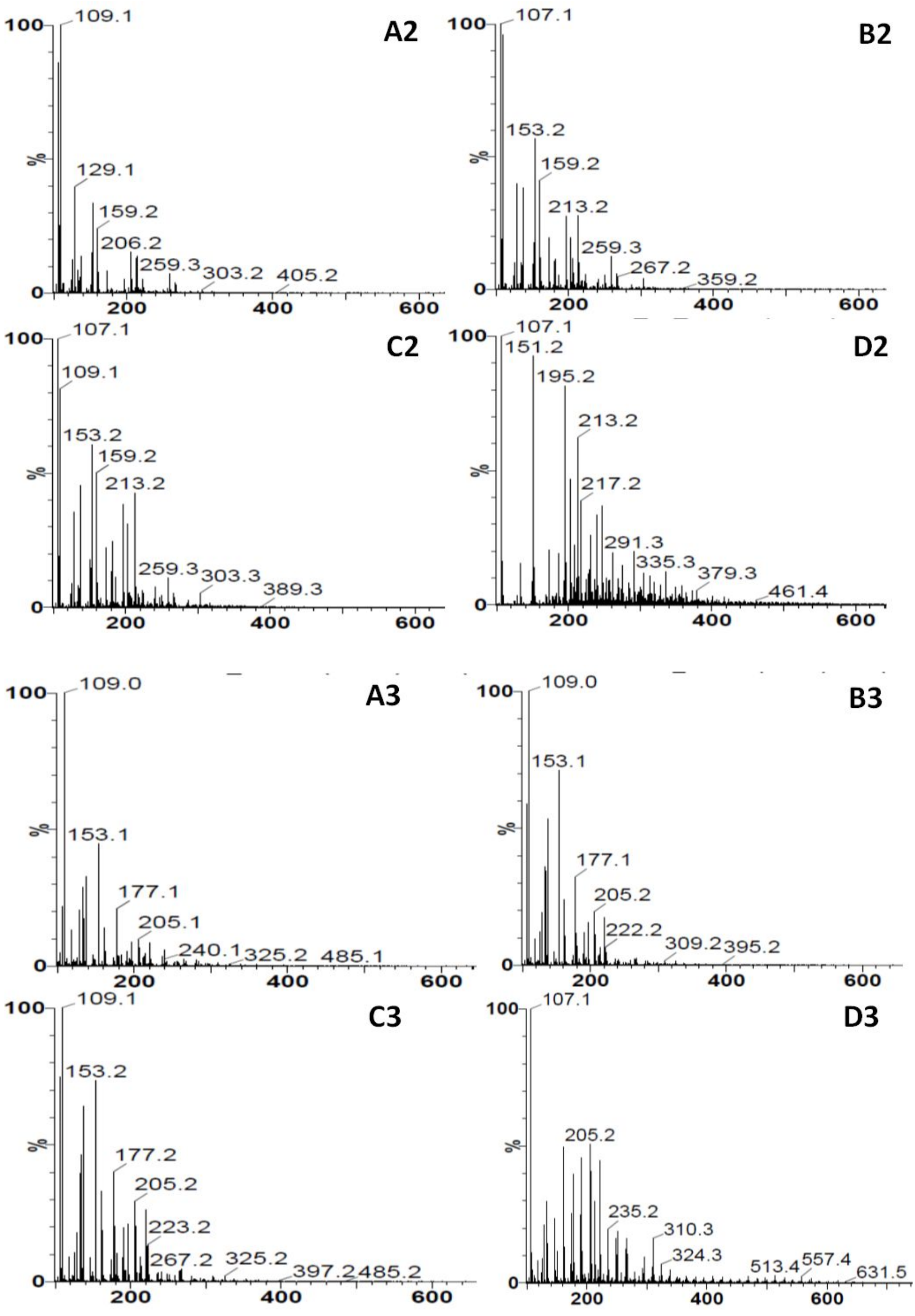

Figure S13. Mass spectra dynamics in LCMS instruments. A,B,C,D are 30min, 2hours, 24hours, and 48hours respectively. The numbers 1,2,3 represent the CDs (m-γ), (p-γ),(o-γ).

3.4 X-ray Photoelectron Spectroscopy

XPS samples were prepared from dried carbon dots placed over carefully cleaned silicon slides. Canning 5600 AES/XPS multi-technique system (PHI, USA) is a state-of-the-art analytical tool for chemical analysis of any solid material, ranging from Li to U. It can determine the chemical composition of surfaces not only by their atomic content but also by the chemical bonding of the surface atoms. In our case, the elemental analysis yielded.

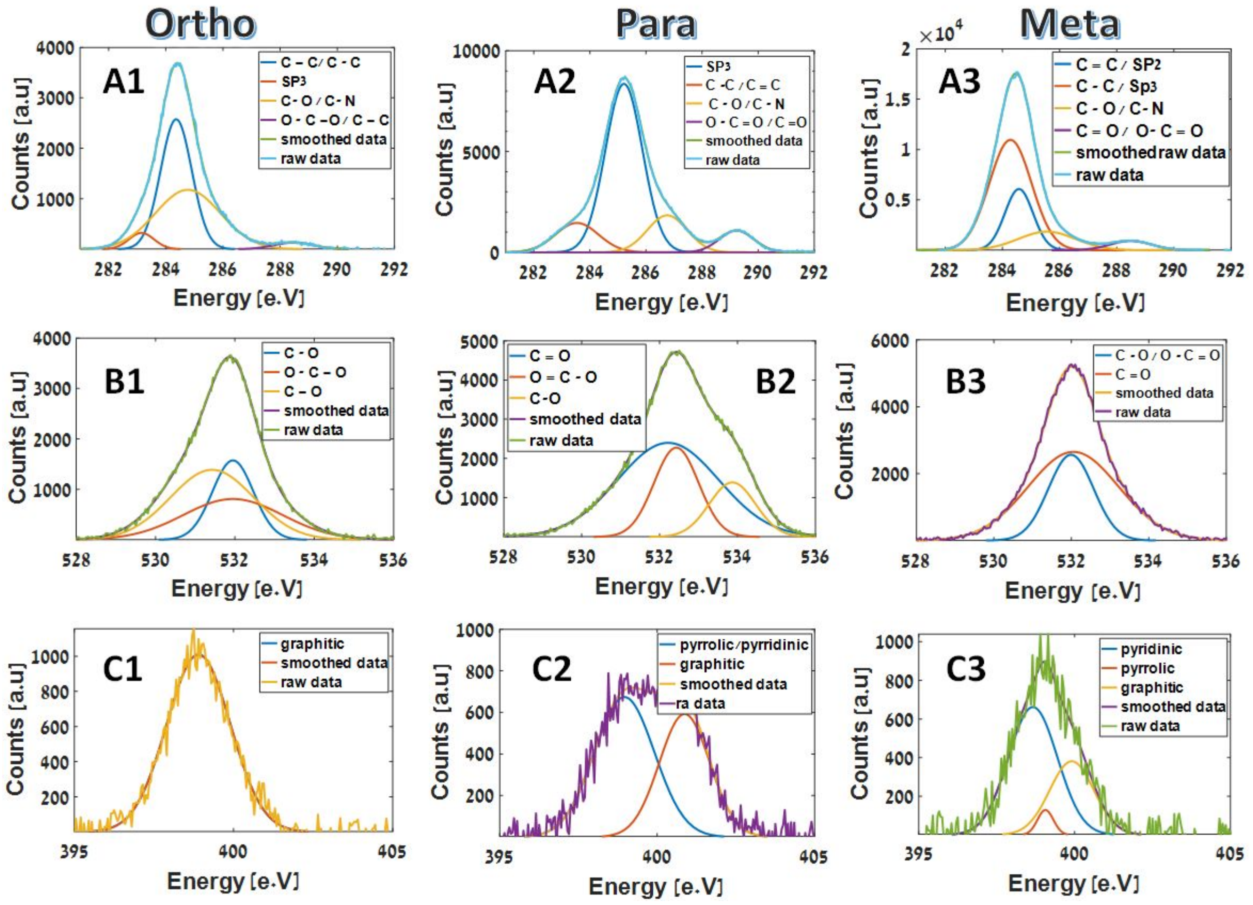

Figure S14. XPS data analysis. A. XPS data of the main bands exist in p-η6CDs structure A1-B1-C1, m-η6CDs structure A2-B2-C2, o-η6CDs structure A3-B3-C3. Peak analysis and Gaussian decomposition fit for A. C1S B. O1S C. N1S

Element	η6pPD [%]	η6oPD [%]	η6mPD [%]	δ3mPD [%]
C	75.41	75.81	78.73	69.48
O	18.72	15.54	15.76	23.15
N	2.23	2.51	0.74	0.36
P	-	-	-	7.01

Table S4. Elemental analysis results from XPS. C, N O elements absolute abundance in percent as obtained from fractionation of XPS energy abundance data.

3.5 Fourier Transform InfraRed (FTIR) Spectroscopy

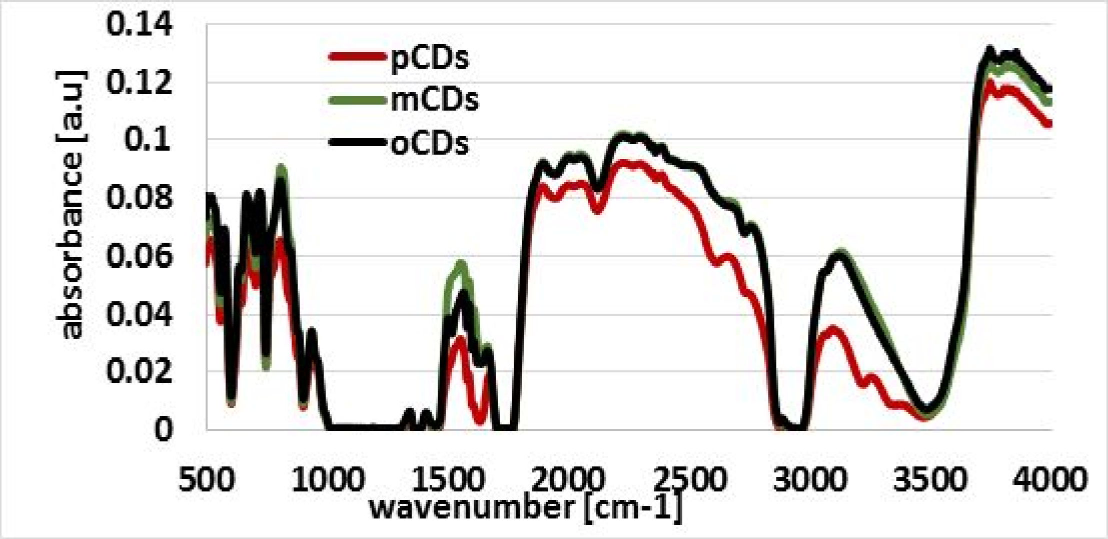

Figure S15. FTIR spectra of CDs at the range 500-4000cm-1. Samples were tested by reflection over silicon slides. All samples were dried from γCDs. Samples are prepared in 2M. For all three isomers. Under 2M HCl initial concentration

6. H^1NMR Hydrogen Nuclear Magnetic Resonance

Dried and purified mCDs powder are dissolved in D_2O. Then inserted to NMR Bruckers Ascennd 500 High Resolution NMR machine. The field was at 500MHz 11.7Tesla. The spectra obtained from sample ***m-η8CDs***.

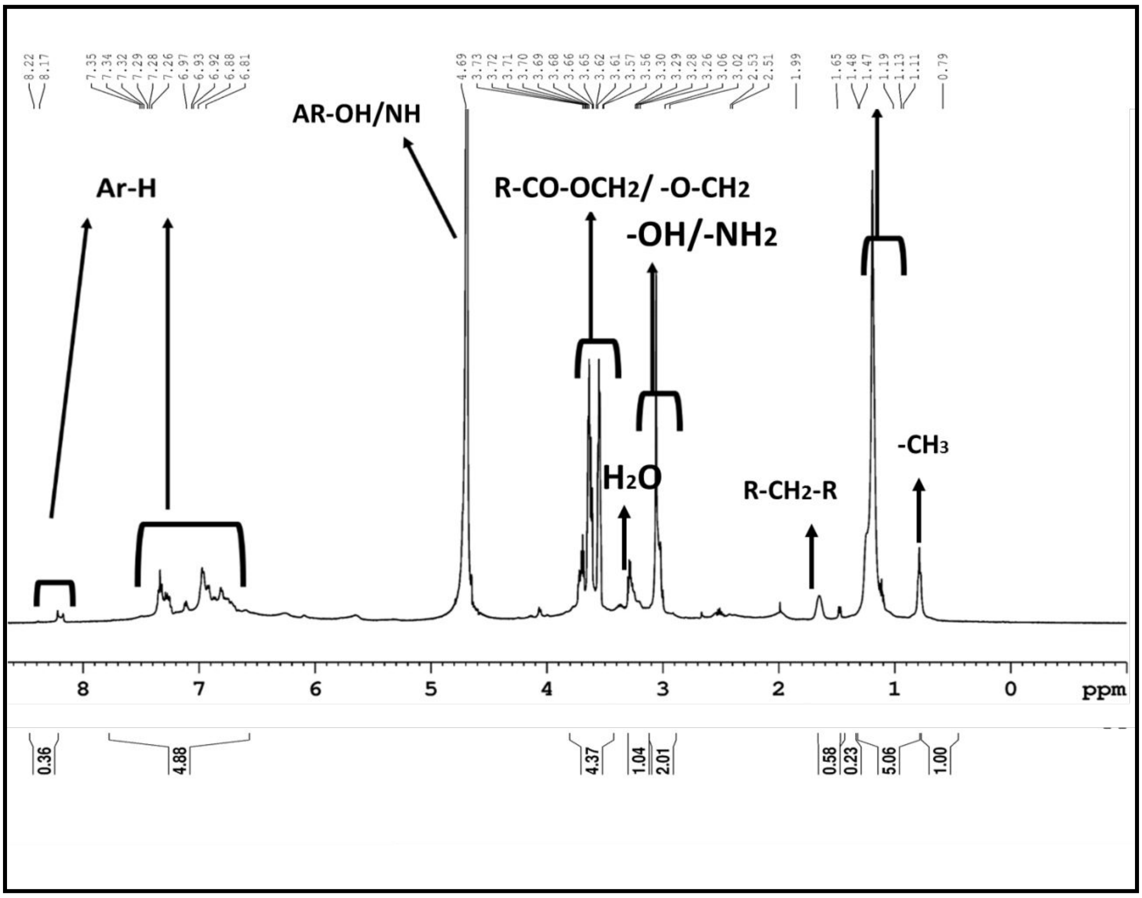

Figure S16. H^1NMR Spectra of m-η8CDs.

3.6 Molecular simulations

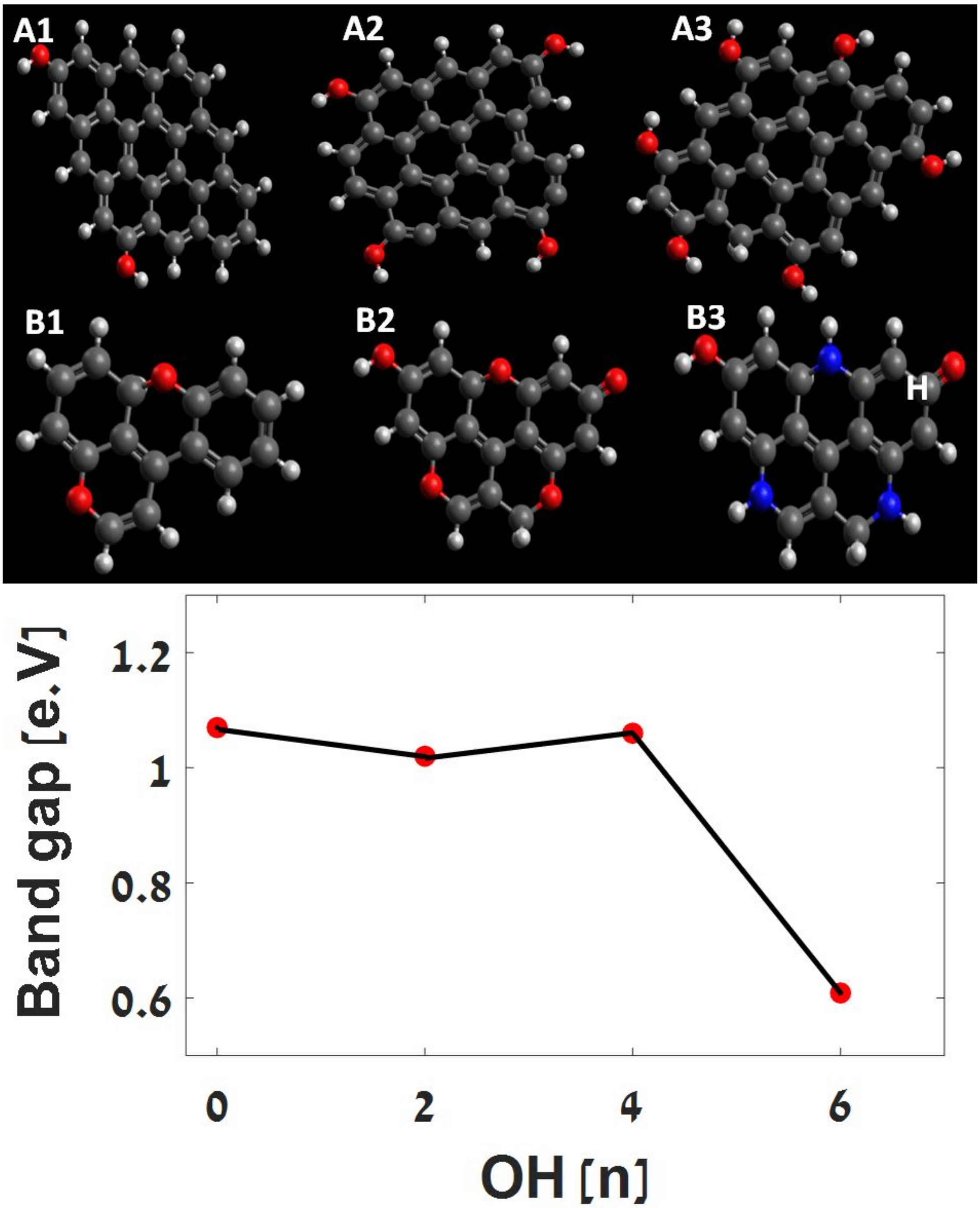

Figure S17. Investigated structures of carbon-based dots. (A1)-(A3) 9 ring CDs with an increased number of hydroxyl groups on its surface. (B1) CD1 from 4 rings (B2) 5 ring CD2 with 5 oxygens (B3) Similar to B2 with nitrogen variation

Structure	Bandgap [eV]
CD1	2.032318
CD2	1.99
CD3	1.558998

Table S5. Bandgap values of CD1-CD3 Fig S14.

4. Carbon dot synthesis kinetics

Our simple model is based on the first order consecutive reactions: According to the model, x refers to the initial isomer of Phenylenediamine (x = ortho, meta, para), x_1 corresponds to the photoactive reaction product of the respective isomer, x_2 corresponds to the non-fluorescent product obtained from x_1 degradation, and a direct formation from PD or other species. That is summarized in the following reaction

$$\boldsymbol{nxPD \ \rightarrow_{k_0} xPD^{\#} \ \rightarrow_{k_1} \ x_1CD \ \rightarrow_{k_2} \ x_2CD}$$

Starting from the nxPD, there are n phenylenediamine x'th isomer, then it transforms into transition state where k_0 is a fast transformation $xPD^{\#}$. The concentrations of the reaction components are;

$$\textbf{1. } [\boldsymbol{x_1CD}]_{\boldsymbol{0}} = [\boldsymbol{x_2CD}]_{\boldsymbol{0}} = \boldsymbol{0} \qquad \textbf{2. } [\boldsymbol{xPD^{\#}}]_{\boldsymbol{0}} = [\boldsymbol{xPD^{\#}}]_{\boldsymbol{t}} + [\boldsymbol{x_1CD}]_{\boldsymbol{t}} + [\boldsymbol{x_2CD}]_{\boldsymbol{t}}$$

solving the set of the three differential equations related to each of the reaction components yields:

$$\frac{d[xPD]}{dt} = -\,k_1[xPD]$$	(1)
$$\frac{d[x_1CD]}{dt} = k_1[xPD] - k_2[x_1CD]$$	(2)
$$\frac{d[x_2CD]}{dt} = k_2[x_1CD]$$	(3)

That set of variables has an analytic solution. Interesting in the x_1CD, the desired fluorescent Carbon dot in all of its divergence in compounds (photoactive), the latter thermally degrades to inactive x_2 forms of the x's isomer. The analytical solution for the active form;

$$(4) \quad [x_1CD]_t = \frac{[xPD]_0 k_1}{k_2 - k_1}[exp\,(-\,k_1t)\; - exp\,(-k_2t)\,]$$

The fluorescence intensity is linked linearly to the concentration of the active CDs. The relation between the FL intensity and concentration is made possible at low concentration. Hence, the sample measurement of tracing the active compounds is in its linear region. More, decreasing to the minimum any effects of shading or quenching. Henceforth, using the analytical solution of the first-order kinetics enabled a full determination of the approximated model. Then, further analyzed to understand the reaction properties. Collision theory implemented and the rate of collisions was estimated then used to calculate the activation energy from the Arrhenius model: The model is summarized in the equation $K = A * exp\,(-\frac{Ea}{RT})$, where **K** - Rate constant, **A** - pre-exponential factor, **Ea** - Activation energy, **R**- Gas constant and **T** - Temperature. According to the collision theory $A = Z_{AB} = N_A\sigma_{AB}\sqrt{\frac{8K_BT}{\pi\mu_{AB}}}$. N_A- Avogadro's number, σ_{AB}: molecular cross-section $\sqrt{\frac{8K_BT}{\pi\mu_{AB}}}$ - Average velocity, K_B - Boltzmann's constant, μ_{AB}- the reduced mass.

Sampling the carbonization evolution is executed by soaking about 1mL from the reaction batch at different time intervals. A glass pipette employed for that purpose is inserted with minimal interference on the reaction. The maximal fluorescence intensity measured from each sample with respect to the maximal excitation wavelength data obtained in different time intervals. The collection is done with minimal interference with the reaction atmosphere as described in Supplementary Information. Data fitting of the max intensity vs. time using MATLAB Simulink software to eq (4) retrieved the desired kinetic parameters. Same fitting properties obtained in both regular fit and with normalized data to the maximal peak value of the intensivist point (Fig S15).

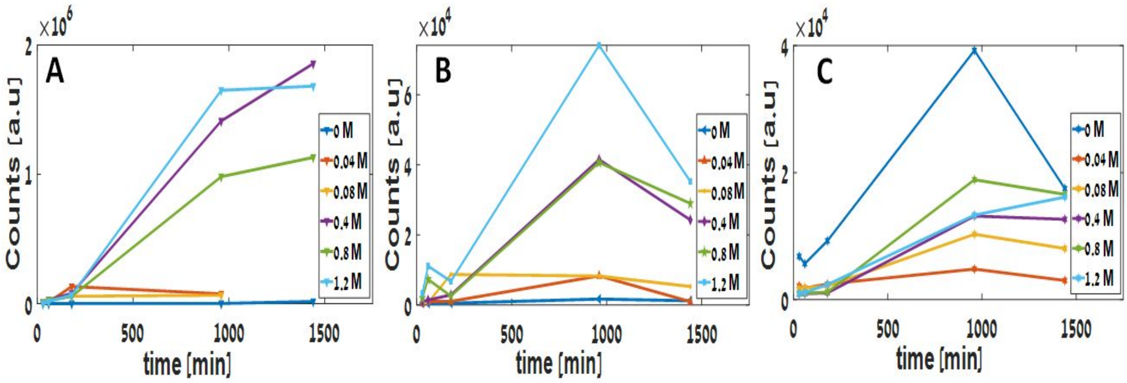

Figure S17. A,B,C. are emission maxima dynamics for the set $\eta 1m - \eta 6m \quad \eta 1o - \eta 6o \quad \eta 1 p - \eta 6p$ with (0 - 0.04 - 0.08 - 0.4 - 0.8 - 1.2) M respectively see the legend.

$Reaction\ \eta_m$	$HCl\ [M]$	$K1$	$K2$	$Reaction\ \eta_o$	$K1$	$K2$	$Reaction\ \eta_p$	$K1$
a	0	7.87E-06	0.02224	a	0.00013	0.007955	a	0.001144
b	0.04	0.000107	0.009986	b	0.000448	0.005866	b	0.000304
c	0.08	5.03E-05	0.007109	c	0.000638	0.004892	c	0.000462
d	0.4	0.000181	0.000283	d	0.001407	0.001321	d	3.32E-05
e	0.8	0.00016	0.000873	e			e	4.90E-05
f	1.2	0.000316	0.0011	f	0.003	0.000689	f	0.000187

Table S6. Rate constants as observed from experimental data and calculated in Matlab.

By applying the estimation values of collisions to the experimental rate constant, obtaining results showed in table S5 for mCDs in different acid concentration:

Reaction	HCl [M]	$k_1[s^{-1}]$	$Ea_{k_1}[\frac{kj}{mol}]$	$k_2\ [\frac{M}{S}]$	$Ea_{k_2}[\frac{kj}{mol}]$
$\delta 9m$	***1.2***	***0.00185***	***120.8±1.6***	***0.00184***	***120.9±1.5***
$\delta 8m$	***0.6***	***0.001182***	***109.5±1.6***	***0.001182***	***123.0±1.6***
$\delta 7m$	***0.12***	***4.29E-05***	***125.8±1.7***	***0.00098***	***119.1±1.6***

Table S7. Activation energies estimation for reaction set $m - \eta 7 - m - \eta 9$ with both constants of formation and degradation approximated to first-order kinetics.

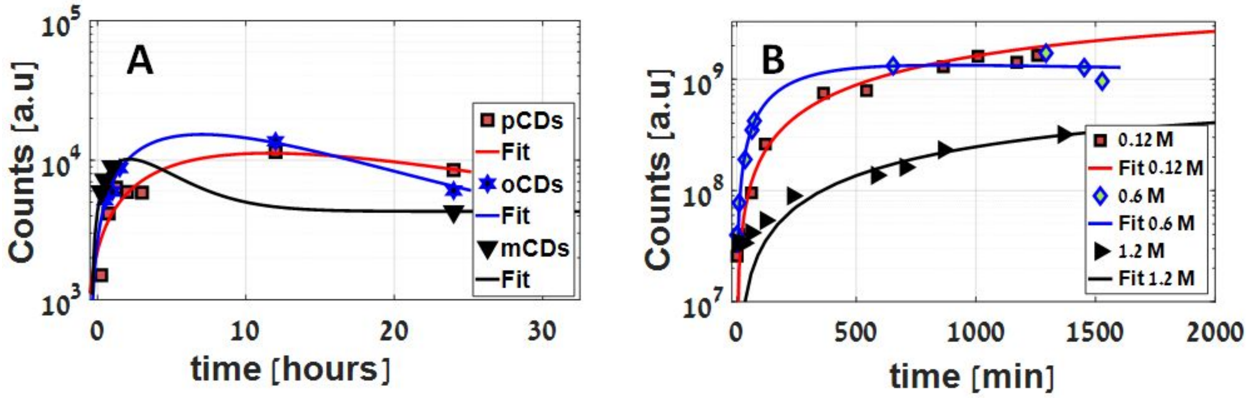

Figure 18. Reaction product fluorescence intensity over reaction time with fitted kinetics for (A) γm, γp, γo different PD isomers (B) $\eta 7m - \eta 9m$ different acid molarities of m-CDs.

5. High-performance liquid chromatography (HPLC)

Samples of 10mg/mL of a standard solution containing mPD in EtOH. The sample was mixed 1/100 v/v with acetonitrile then centrifuged for 5min. Then the sample runs through the C18 column with KH2PO4.

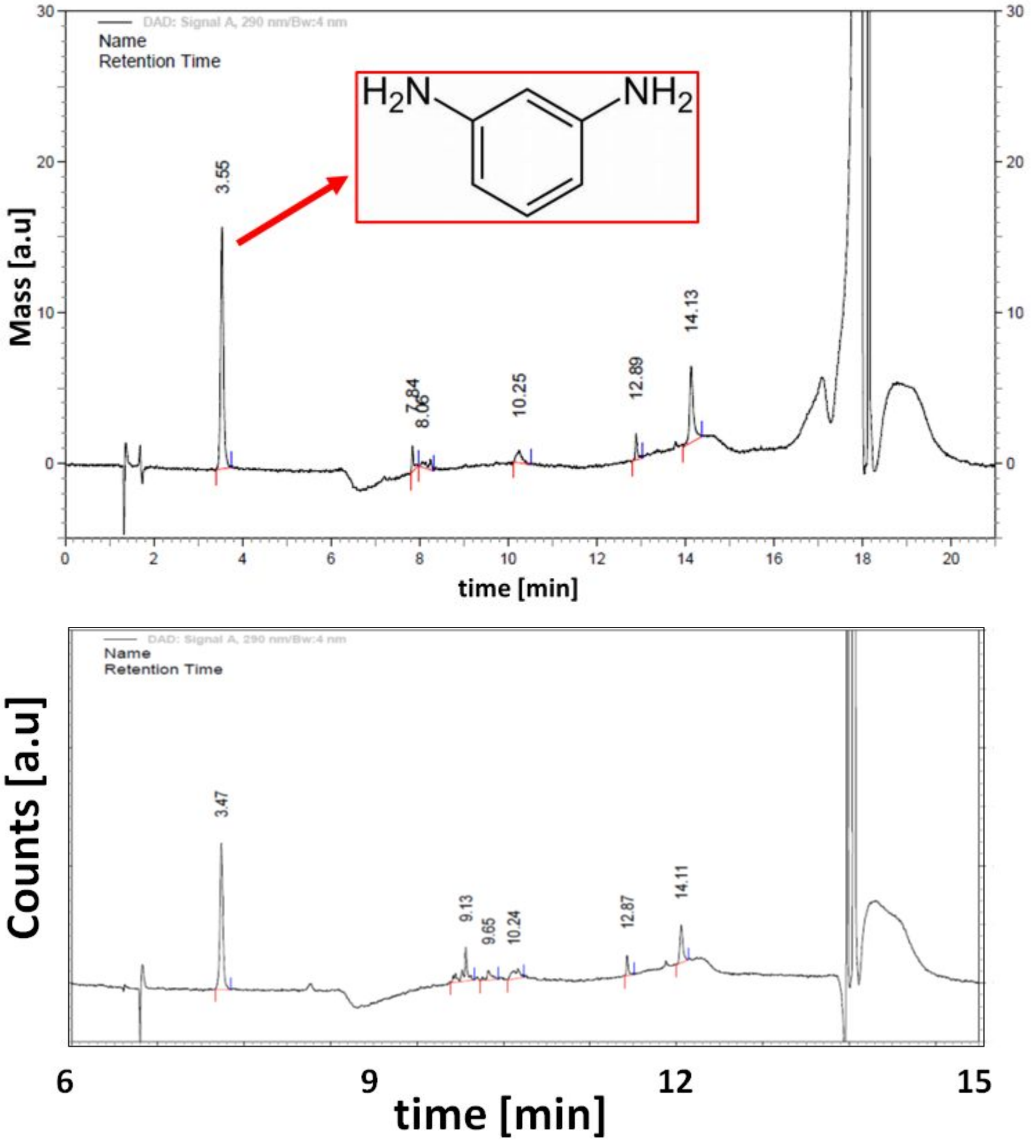

Figure S19. (A) One of the standard chromatograms for mPD, the area marked at 3.55min is the mPD. (B) m-η8CDs after 60min

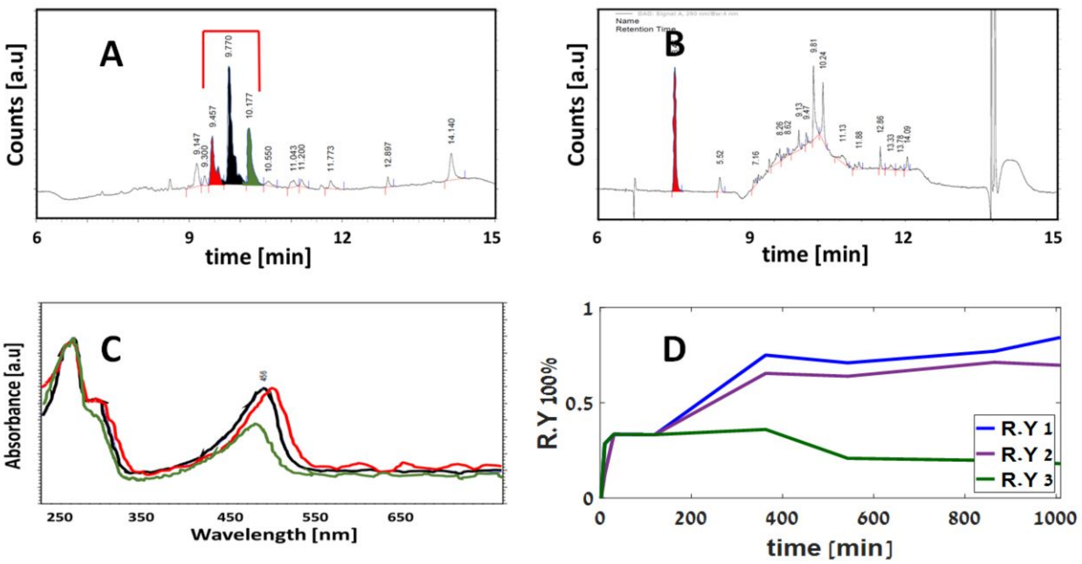

Figure S20. (A) HPLC chromatogram of m-η8CDs samples extracted from the reaction after ~16hours, the red marked area represents the photoactive CDs that hit the detector at 9.45 9.77 10.25 minutes (B) HPLC chromatogram of m-η8CDs sample extracted from the reaction after ~6hours with the addition of mPD (C) Absorbance of the three HPLC fractions at (A) red, black, and green are 9.45 9.77 10.25 minutes respectively (D) in terms of fraction from the mixture, RY1 corresponds to overall (blue line) corresponds to fragments that maintain the same absorbance spectra RY 2(purple) corresponds to brightest fractions only, RY 3 (green) is the abundance of the brightest fraction at 10.25minutes

7. Quantum yield measurements

In order to calculate the relative QY of the resulting product, different references were used and then an averaged value was introduced, the main overlapping spectra is that of fluorescein thus, used as the primary reference. Fluorescein QY in 0.1M NaOH was referenced to that of Rhodamine 6G in pure was used to cross corelate the $\frac{n_x}{n_r} \cdot \frac{A_x}{A_r} \cdot \emptyset_r = \emptyset_x$Ethanol. The relation - is the $\frac{n_x}{n_r}$ is the QY under study is a reference QY, and $\emptyset_r$, $\emptyset_x$references. – are the absorbance of the A_r ,A_x .ratio of refractive indices of the solvents measured sample and the reference sample respectively. Fluorescein QY Value appeared to be 91%.

The gradient method that averages over a range of concentration is also used to obtain statistical data to increase the accuracy of QY measurements.
The quantum yield was fixed for 550nm excitation to 100% then the QY value at 420-450nm was the intensity percentage of the 550nm excitation.

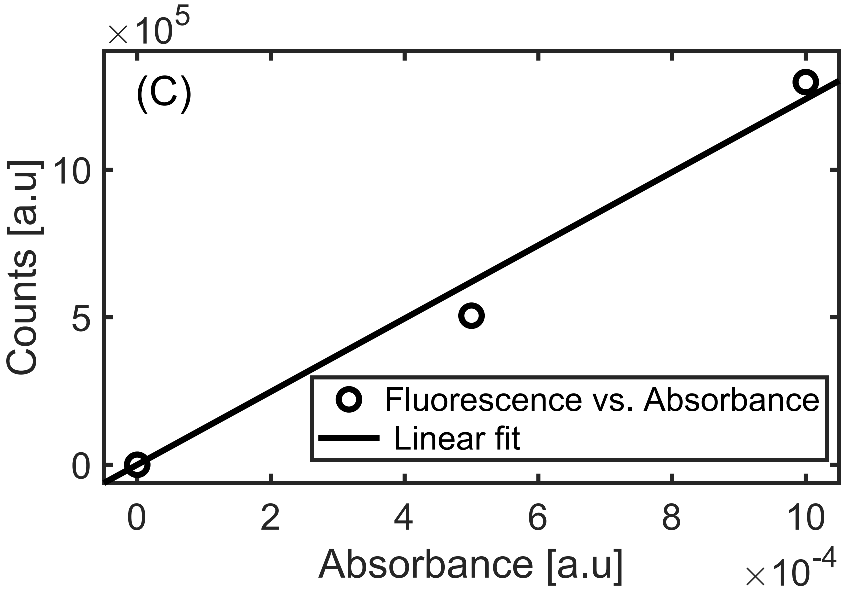

Figure S21. Fluorescence vs absorbance calibration curve of Fluorescein dye in 0.1M NaOH, the slope is used as a reference for calculations of QY considering this one as 90%.

Material	*Preparation method*	*reference*	*QY 450*	*method checked*	*QY 425*	*QY 420*	*method checked*
mPD con1	plate	Fluorescein water at 450	0.370	slope	0.470		
mPD HCl D	reflux	Fluorecein water at 450	0.200	slope			
mPD 1500HCL 24h	plate	Fluorescein water at 450	0.160	slope	0.340		
mPD HCL a	reflux	Fluorescein water at 450	0.010	one point		0.010	one point
mPD HCL b	reflux	Fluorescein water at 450	0.190	one point		0.180	one point
mPD HCl c	reflux	Fluorecein water at 450	0.180	one point	0.190		
MPD 0.5mL HCl	plate	Fluorescein water at 450			0.37		

Table S8. QY values of different CDs as measured from fluorescein reference.
